# Androgen enhances the activity of ETS-1 and promotes the proliferation of HCC cells

**DOI:** 10.18632/oncotarget.22669

**Published:** 2017-11-25

**Authors:** Hui Ren, Bo Ren, Jiabin Zhang, Xiaofeng Zhang, Lixin Li, Lingzhan Meng, Zhijie Li, Jia Li, Yinjie Gao, Xuemei Ma

**Affiliations:** ^1^ Liver Transplantation and Research Center, 302 Hospital, Beijing 100039, China

**Keywords:** androgen, androgen receptor, ETS-1, HCC, proliferation

## Abstract

The expression of androgen receptor (AR) has been detected in hepatocellular cancer (HCC). However, there is no universal model detailing AR’s function and mechanism in HCC. This study’s results show that treatment with dihydrotestosterone (DHT), an endogenous androgen, promoted HCC cells’ proliferation and up-regulated the transcription factor activity of ETS-1 (E26 transformation specific sequence 1), which mediates the migration and invasion of cancer cells via protein-protein interaction between AR and ETS-1. Results from luciferase assays showed that ETS-1’s activity was significantly up-regulated following androgen treatment. AR mediated ETS-1’s DHT-induced transcription factor activity. A potential protein-protein interaction between ETS-1 and AR was identified via glutathione S-transferase (GST) pull-down and co-immunoprecipitation assays. The mechanisms’ data indicated that enhancing AR activity increases ETS-1’s activity by modulating its cytoplasmic/nuclear translocation and recruiting ETS-1 to its target genes’ promoter. Moreover, while overexpression of AR significantly increased the proliferation or *in vitro* migration or invasion of HepG2 cells in the presence of androgen, inhibiting AR’s activity reduced these abilities. Thus, AR’s function as a novel ETS-1 co-activator or potentially therapeutic target of HCC has been demonstrated.

## INTRODUCTION

In mammalian cells, androgen responds to androgen receptor (AR), which plays a central role in male health and the maintenance or progress of prostatic carcinoma [1]. AR, a member of the nuclear receptor protein superfamily, contains four major functional regions: N-terminal transactivation domain (NTD), DNA-binding domain (DBD), C-terminal ligand-binding domain (LBD), and hinge region [2]. In cell nuclei, AR binds to the androgen responsive element (ARE) to regulate the transcription of genes responding to the androgen [3]. Researchers have identified that androgen/AR plays a key role in prostatic carcinoma’s maintenance and development [1–3]. It may also be involved in other types of human cancer. Tian et al. summarized the research of AR in HCC and indicated that AR participates in HCC progress [4]. Chen et al. reported that AR enhances the proliferation of HCC cells by suppressing tumor suppressors [5]. Although multiple studies have shown that AR is involved in HCC’s progression [6–10], the function and detailed mechanisms of how AR regulates HCC’s cell proliferation remain unclear. A deeper understanding of how AR regulates the proliferation, migration, and invasion of HCC cells will be helpful for developing further treatments.

The transcription factor ETS-1, which belongs to the ETS protein family, contains the ETS (transcription activation domain) and helix DNA-binding domains [11]. In the nucleus, ETS-1 binds to the ETS-binding elements (EBS) 5’-GGAA/T-3’ in the promoters/enhancers of the targeted genes (e.g., matrix metallopeptidase (MMP)1 or MMP9) and in turn mediates the proliferation, development, metastasis, invasion, and angiogenesis of human cancerous cells [12]. A high level of ETS-1 protein is associated with poor breast cancer prognosis [13]. The transcriptional activity of ETS-1 is regulated by some co-regulators, such as SRCs (steroid receptor co-activators) and AIB-1 (amplified in breast cancer 1) [13]. It is valuable to identify the novel co-factors or mechanisms involved in the regulation of ETS-1’s transcriptional activity. Massie et al. uncovered a potential interaction between ETS-1 and AR at a subset of AR promoter targets by using chromatin immunoprecipitation with on-chip detection of genomic fragments in prostate cancer cells [14]. It was also reported that AR promotes the migration and invasion of upper urinary tract urothelial carcinoma cells by up-regulating MMP-9 and cyclooxygenase-2 (COX-2) [15]. Previous evidence has also demonstrated that some transcription factors or nuclear receptors may cross-talk in a feedback way [16]. For example, the aryl hydrocarbon receptor (AHR) can regulate the ER (estrogen receptor) signaling pathway through protein-interaction, and the ER can also repress the AHR target genes’ transcription [16]. The same mechanisms were also identified between a nuclear factor of activated T-cells 3 (NFAT3) and ER [16]. Zhang et al. also showed that high levels of AR and matrix MMP 2/9 in HCC clinical specimens compared with adjacent non-tumor tissues were predictors of invasion and staging [17]. Thus, examining whether AR modulates ETS-1’s activity in HCC cells was chosen for this research.

HCC is the most common type of human cancer [18]. Despite new insights and advances in therapeutic strategies, the general prognosis for HCC patients remains poor [18, 19]. Thus, identifying novel targets of HCC is vital. This study’s results prove that AR interacts with ETS-1 in HCC cells. ETS-1’s activity increased significantly when AR was activated by its ligand, DHT (dihydrotestosterone). Also, enhancement of AR activity via androgen significantly promoted HCC cell proliferation *in vitro* and *in vivo*.

## RESULTS

### Androgen enhances the transcription factor activity of ETS-1 and the expression of ETS-1 response genes

**Figure 1:**
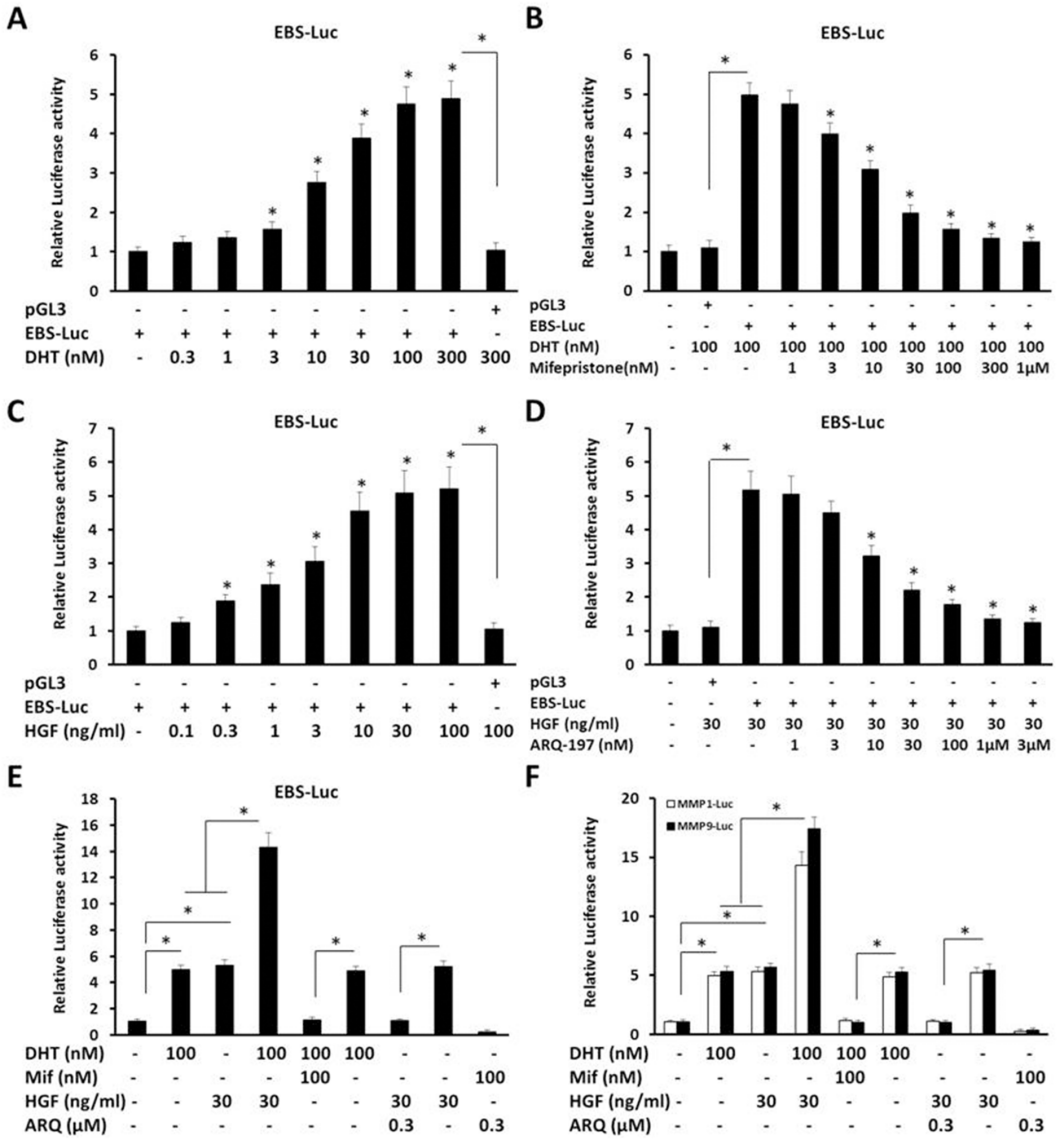
The dose-effect of androgen, mifepristone, HGF, or ARQ-197 on the transcription factor activity of ETS-1 **(A-F)** HepG2 cells, which were co-transfected with EBS-Luc, MMP1-Luc, or MMP9-Luc reporters, were treated with the indicated concentration (A, B, E, F) of DHT (the agonist of AR), (B, E, F) of mifepristone (the antagonist of AR), (C, D, E, F) of HGF (hepatocyte growth factor, the agonist of c-Met) or (D, E, F) of ARQ-197 (the antagonist of c-Met).Then, the cells were harvested and determined by Luciferase assays. The values are the mean ± SD from triplicate independent experiments. ^*^P < 0.05.

To discover whether androgen modulates the transcription factor activity of ETS-1, luciferase assays were performed in HepG2 cells, which were co-transfected with ETS-1 binding site EBS-Luc reporters. As shown in Figure 1 and Table 1, DHT, a common endogenous androgen, increased the ETS-1 activity in a dose-dependent manner (Figure 1A); the *EC*_*50*_ value was 28.61 ± 4.75 nM. At the same time, the antagonist of AR, mifepristone, down-regulated the activity of EBS-Luc induced by DHT (Figure 1B); the *IC*_*50*_ value was 17.12 ± 2.44 nM. Next, to further test the activity of endogenous ETS-1 in HepG2 cells, the agonist hepatocyte growth factor (HGF) and antagonist tivantinib (ARQ-197) of the ETS-1 signaling pathway were used. As shown in Figure 1C and 1D, while HGF induced the EBS-Luc reporter activity in a dose-dependent manner (*EC*_*50*_ value = 7.55 ± 1.02 ng/ml), ARQ-197 inhibited this activity (*IC*_*50*_ value = 20.44 ± 2.95 nM). Together, these results indicate that enhancing AR activity increased ETS-1transcriptional activity.

**Table 1:** The dose-effect of agents on ETS-1’s transcriptional activity

Agents	*IC*_*50*_/*EC*_*50*_ (nM)	*IC*_*max*_/*EC*_*max*_ (μM)	*R*^*2*^ Value	*P* Value
**DHT**	28.61±4.75	0.10	0.91	0.00092
**HGF**	7.55±1.02 (ng/ml)	0.03	0.95	0.0011
**Mifepristone**	17.12±2.44	0.10	0.92	0.0086
**ARQ-197**	20.44±2.95	0.30	0.91	0.0024

Then, the potential cross-talk of AR and ETS-1 signaling pathways was detected. HepG2 cells, which were co-transfected with EBS-Luc, or ETS-1 targeted genes MMP1/9’s luciferase reporters MMP1-Luc or MMP9-Luc, were harvested and analyzed by luciferase assays. As shown in Figure 1E and 1F, DHT and HGF synergistically up-regulated the activity of EBS-Luc, MMP1-Luc, and MMP9-Luc. Mifepristone inhibited the effect of DHT but not HGF; whereas ARQ-197 almost blocked HGF’s effect but not DHT.

**Figure 2:**
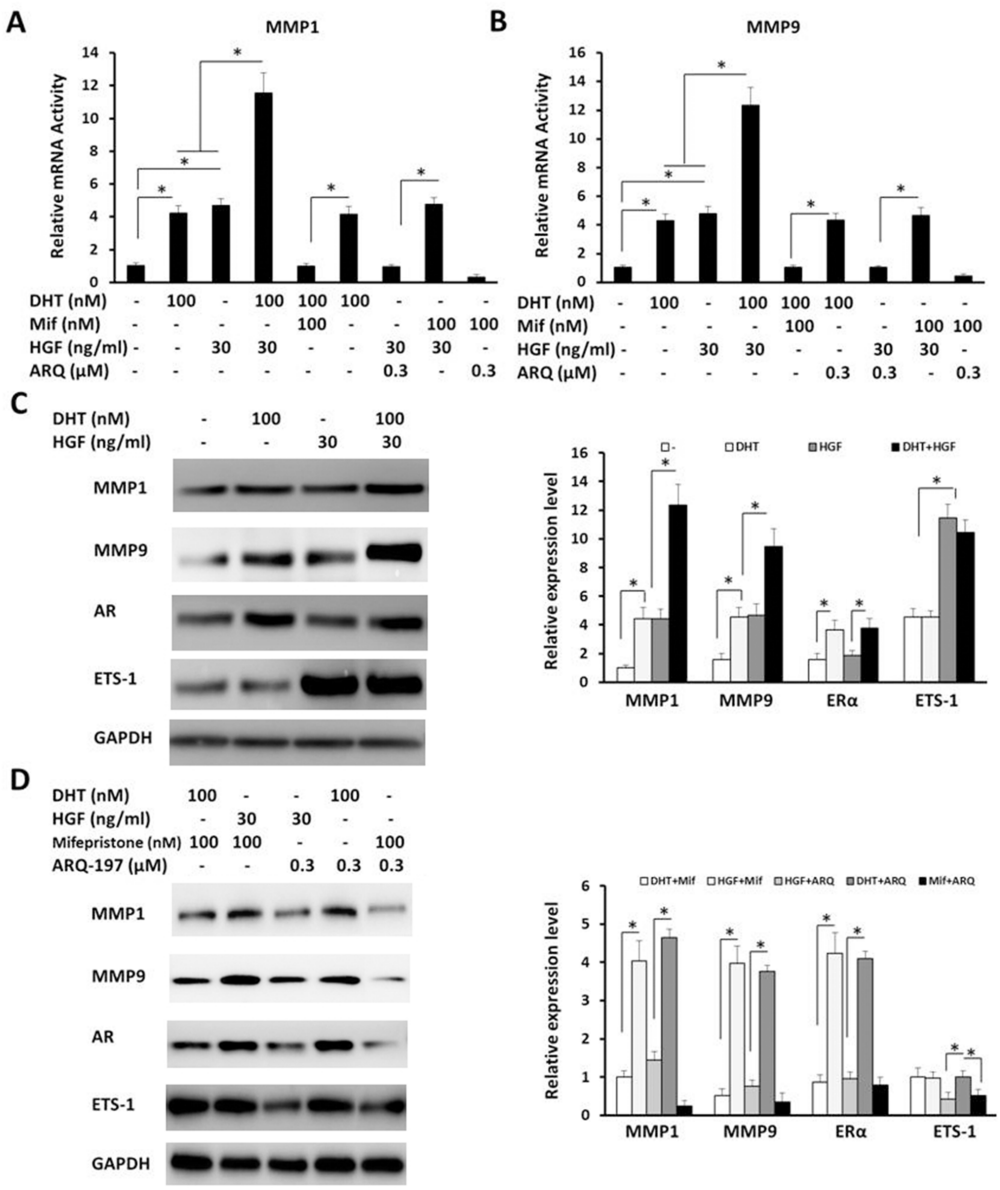
The effect of AR on the expression of ETS-1-targeted genes HepG2 cells were treated with the indicated concentration (*EC*_*max*_/*IC*_*max*_ concentration) of DHT **(A–D)** mifepristone (A, B, D), HGF (A-D), or ARQ-197 (A, B, D). (A–B) Identification of ETS-1-targeted genes’ mRNA level was determined by real-time RT-PCR assays. (C-D) The protein level of AR, ETS-1, or its responses genes were identified by WB assays. GAPDH was used as the loading control. The values are the mean ± SD from triplicate independent experiments. ^*^P < 0.05.

Next, AR’s effect on the expression of ETS-1 response genes was determined by reverse transcription polymerase chain reaction (RT-PCR) and immunoblotting assays. As shown in Figure 2, DHT and HGF synergistically increased the mRNA level (Figure 2A and 2B) and protein level (Figure 2C and 2D) of MMP1 and MMP9. Mifepristone blocked the effect of DHT but not HGF, while ARQ-197 inhibited the effect of HGF but not DHT. Moreover, Mifepristone did not affect the activity of HGF, and the antagonist of these two pathways synergistically reduced the expression of ETS-1 response genes. These results indicate that enhancement of AR activity may up-regulate the expression of ETS-1 targeted genes independent of HGF/c-Met.

### The specificity of androgen functions in ETS-1’s activity

**Figure 3:**
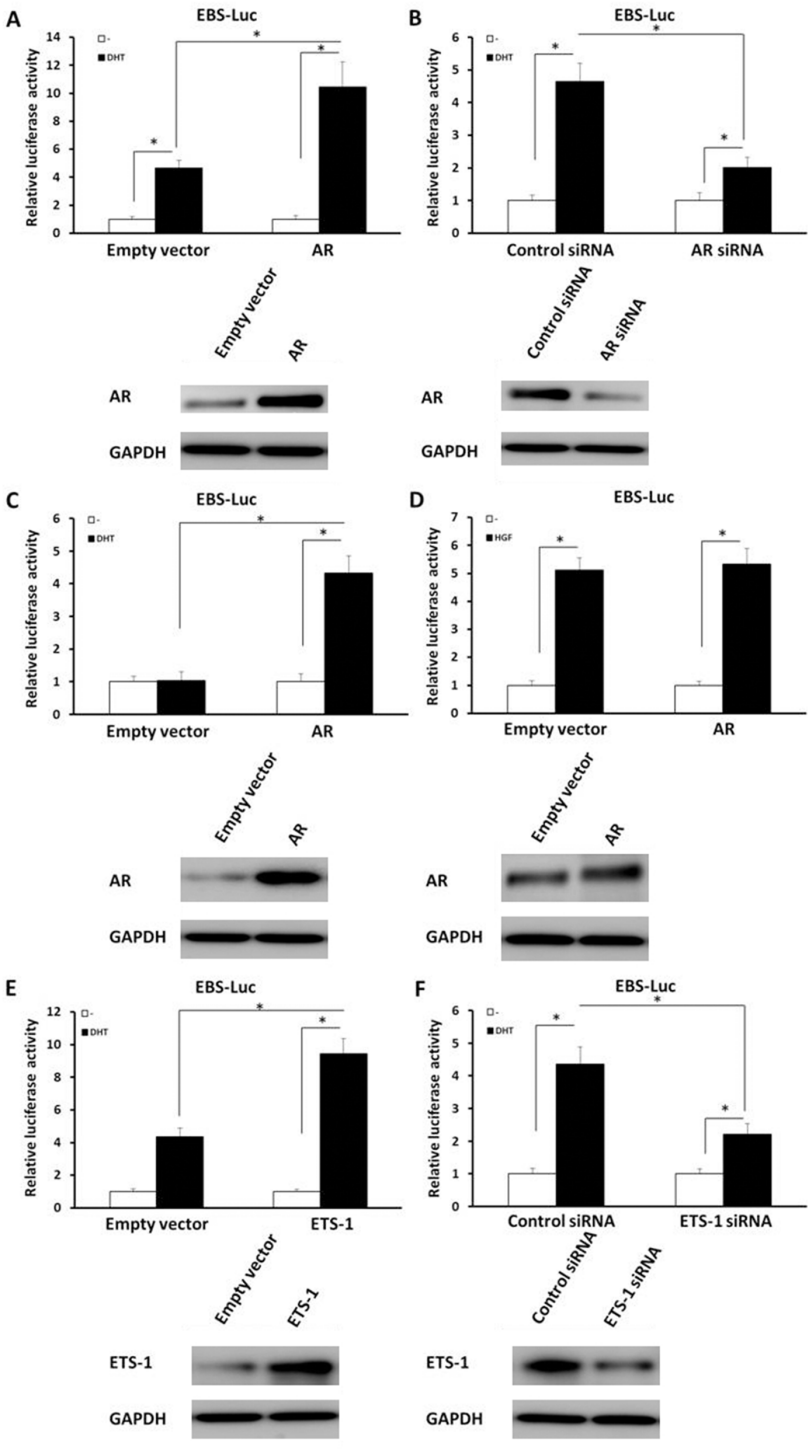
AR (but not HGF/c-Met) mediates the enhancement of androgen-induced ETS-1 activity Cells were treated with 100 nM of DHT **(A-C, E, F)** or 30 ng/ml of HGF **(D)**. HepG2 cells were stably transfected with empty vector (A, D, E), AR vectors (A, D), control siRNA (B, F), AR siRNA (B), ETS-1 vector (E), or ETS-1 siRNA (F), while PC-3 cells were stably transfected with empty vector (C) or AR vectors (C). Then, cells which were co-transfected with EBS-Luc reporters and harvested for Luciferase analysis. The expression of AR and ETS-1 were determined by immunoblots, and the results are shown in the panels at the bottom of the figure. The values are the mean ± SD from triplicate independent experiments. ^*^P < 0.05.

To determine the specificity of DHT’s effect on ETS-1, HepG2 cells were used in co-transfection experiments. To investigate the role of endogenous AR in ETS-1 mediated transcription, HepG2 cells (Figure 3A and 3B and Supplementary Figure 1) were stably transfected with an empty vector, AR vector, control siRNA, or AR siRNA. Overexpression of AR enhanced the activity of EBS-Luc reporter activity only in the presence of DHT (Figure 3A and Supplementary Figure 1). The activity of the EBS-Luc reporters activated by DHT was dramatically reduced in the attenuation of the endogenous AR’s (Figure 3B and Supplementary Figure 1) protein level via AR siRNA compared with the control siRNA group. These data indicate that AR itself is required for the activity of ETS-1’s transcription factor activity induced by DHT.

To further determine whether the observed effects of androgen on ETS-1 transactivation were specific to endogenous AR in HepG2 cells, an AR-negative cell line was used. Human prostate cancer PC-3 cells, which are AR negative and ETS-1 positive, were co-transfected with EBS-Luc, AR vector, or empty vector. As shown in Figure 3C and Supplementary Figure 1, in the presence (but not absence) of DHT, stable expression of AR (but not empty vector) enhanced the activity of EBS-Luc for 4.2-folds. Because androgen may induce ETS-1’s activity in an AR-independent manner, the transcription factor activity of AR in HepG2 was also examined. The results depicted in Supplementary Figure 2 (supplementary data) demonstrate that DHT induced the activity of androgen response element-luciferase (ARE-Luc) reporters’ activity in a dose-dependent manner, whereas mifepristone, the antagonist of AR, disrupted the DHT-induced transcriptional activity of AR. These results reconfirm the fact that AR mediated the DHT-induced transcriptional activity of ETS-1.

Next, ETS-1 signaling’s involvement in AR-mediated transcription was examined. HepG2 cells, which were co-transfected with EBS-Luc, were cultured in Dulbecco’s modified Eagle’s medium (DMEM) supplemented with 0.5% charcoal-stripped fetal bovine serum (FBS) added with or without HGF and analyzed by luciferase assays. As shown in Figure 3D and Supplementary Figure 1, in HepG2 cells, overexpression of AR or its siRNA did not affect the ETS-1’s HGF-induced activity. Then, the effects of ETS-1’s expression on EBS-Luc activity in response to DHT were tested. While overexpression of ETS-1 increased the activity of EBS-Luc (Figure 3E and Supplementary Figure 1) in the presence of DHT, this DHT-activated activity decreased dramatically in the down-regulation of endogenous ETS-1’s (Figure 3F and Supplementary Figure 1) protein level via its siRNA. Collectively, these results indicated that the DHT-induced transcriptional activity of ETS-1 was specifically mediated by AR. Androgen/AR increases the participation of ETS-1 downstream genes (such as MMP1/9) in the invasion of migration of cancerous cells in an ETS-1-dependent manner.

### AR interacted with ETS-1 in an androgen-dependent manner

**Figure 4:**
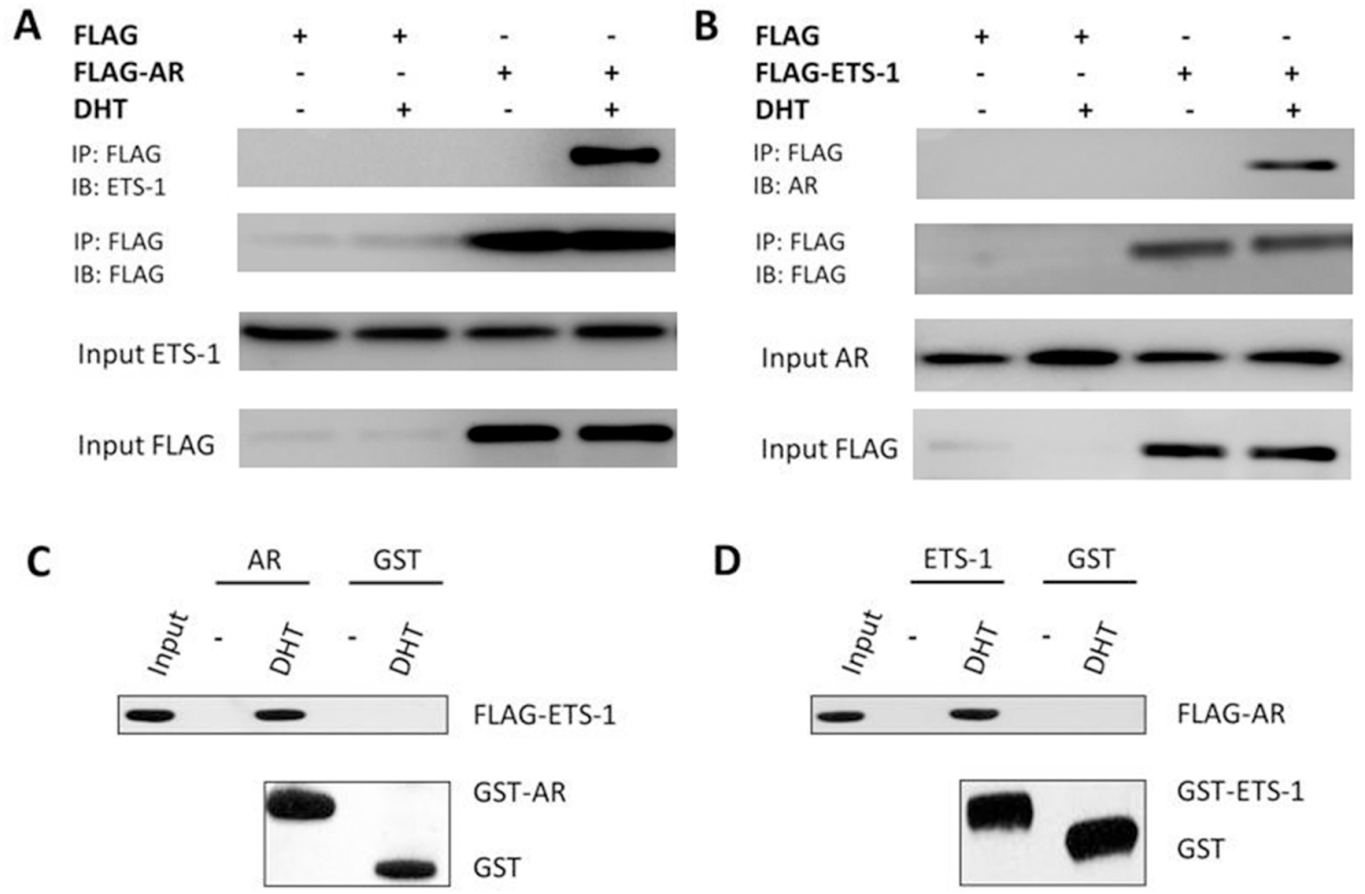
AR can interact with ETS-1 **(A–B)** The interaction of endogenous AR or ETS-1 with exogenous FLAG-ETS1 or FLAG-AR. FLAG-tagged AR (A), FLAG-tagged ETS-1 (B), or FLAG empty vector (A–B) was transfected into HepG2 cells. Cell lysates were immunoprecipitated by an anti-FLAG monoclonal antibody, and the precipitates were then immunoblotted with anti-ETS-1 or anti-AR antibody. **(C–D)** *In vitro* interaction between ETS-1 and AR. Glutathione-Sepharose beads bound with GST-AR (C), GST-ETS1 (D), or GST (C–D) were incubated with purified FLAG-labeled ETS-1 or AR in the presence or absence of 100 nM DHT. After washing the beads, the bound proteins were eluted and subjected to SDS-PAGE and IB assays.

Next, the possible interaction between ETS-1 and AR was investigated. HepG2 cells were transfected with FLAG-AR or FLAG empty vector. Then the co-immunoprecipitation (co-IP) and immunoblotting (IB) assays were performed. The results shown in Figure 4 suggest that FLAG-AR interacted with the endogenous ETS-1 (Figure 4A, Supplementary Figure 3) in a ligand-dependent manner. Then, the re-IP (re-immunoprecipitation) assay was performed. FLAG-ETS1 also interacted with endogenous AR (Figure 4B, Supplementary Figure 4) in the presence of DHT.

Because AR may bind to ETS-1 indirectly, learning whether ETS-1 interacts with AR *in vitro* is valuable. The purified GST-AR or GST-ETS1 was incubated with purified FLAG-ETS1 or FLAG-AR for GST pull-down. A protein-protein interaction between GST-AR with FLAG-ETS1 (Figure 4C and Supplementary Figure 5) or GST-ETS1 with FLAG-AR was detected (Figure 4D and Supplementary Figure 5). Taken together, these observations indicate that ETS-1 potentially binds to AR in a ligand-dependent manner and suggest that androgen enhances ETS-1’s activity by inducing AR/ETS-1 interaction.

### Effect of androgen on ETS-1’s cytoplasm/nuclear translocation

**Figure 5:**
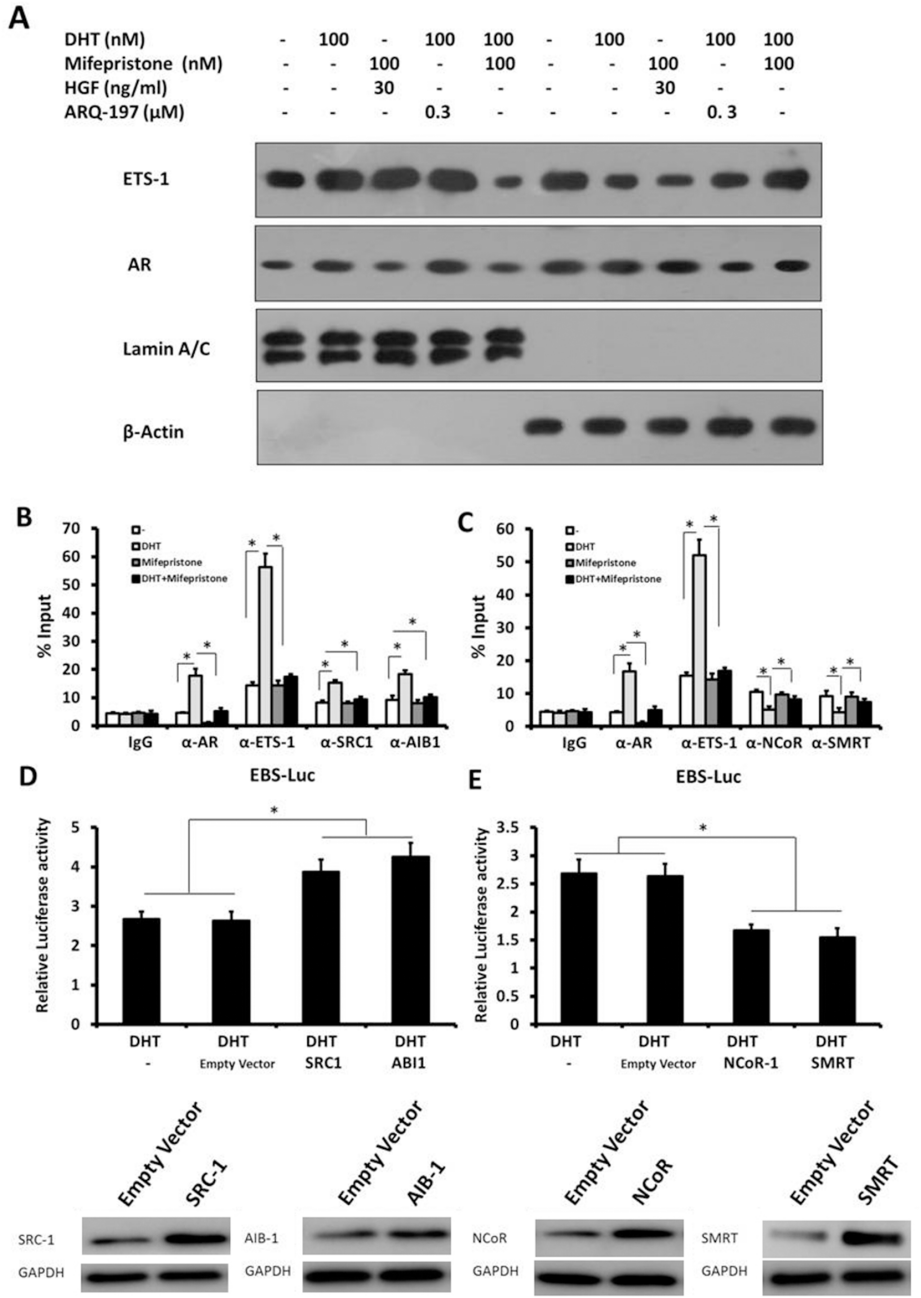
The mechanisms of AR’s effect on ETS-1’s activity **(A)** HepG2 cells were treated with the indicated amount of DHT, mifepristone, HGF, or ARQ-197. Then, cells were fractionated into cytoplasmic and nuclear fractions. The fractions were detected with ETS-1 and ERα antibodies. Lamin A/C was the nuclear indicator. ß-actin was the cytoplasmic marker. **(B)** The recruitment of ETS-1, AR, SRC-1, and AIB-1 to the *mmp1* promoter was detected by ChIP assay. **(C)** The recruitment of ETS-1, AR, NCoR, and SMRT to the *mmp1* promoter was detected by ChIP assay. **(D–E)** HepG2 cells were stimulated with 100 nM DHT for 24 h. Cells were transfected with SRC-1 (D), AIB-1 (D), NCoR (E), or SMRT (E) expression vectors or empty vectors. Cells were then harvested for the luciferase assay. The values are the mean ± SD from triplicate independent experiments. Western blot (bottom) indicates the expression level of the proteins with anti-SRC1, anti-AIB1, anti-NCoR, or anti-SMRT antibodies. GAPDH was used as the loading control. ^*^P < 0.05.

To corroborate the protein-protein interaction results, a subcellular fraction was performed. HepG2 cells, which were treated with agents, were separated into cytoplasmic/nuclear subcellular fractions. As shown in Figure 5, ETS-1 or AR was detected in both the cytoplasmic and nuclear fractions. DHT increased the proportion of AR and ETS-1 in the nucleus (Figure 5A and Supplementary Figure 6). The antagonist of AR, but not the inhibitor of ETS-1 signaling, disrupted the DHT-induced cytoplasmic/nuclear translocation of AR and ETS-1 (Figure 5A and Supplementary Figure 6). These results concur with previous findings that AR regulates ETS-1 activity by altering its cytoplasmic/nuclear translocation dependent to DHT but not its ETS-1 signaling pathway.

### Effect of AR on the recruitment of ETS-1 to targeted gene’s promoter

To further investigate the mechanisms of androgen/AR on ETS-1 chromatin immunoprecipitation (ChIP) assays were performed. The recruitment of ETS-1 and its co-factors to the MMP1 (Figure 5) promoter sequence, which contained the EBS, was detected by the ChIP assay. As expected, AR, nuclear receptor corepressor (NCoR), silencing mediator for retinoid and thyroid receptors (SMRT), ETS-1, SRC-1, and AIB-1 were recruited to the MMP1 promoter (Figure 5B and 5C). DHT promoted the recruitment of AR, ETS1, SRC-1, and AIB-1, while mifepristone down-regulated these recruitments (Figure 5B). At the same time, DHT reduced the recruitment of NCoR and SMRT (Figure 5C), which are transcriptional negative co-regulators of ETS-1. Next, the involvement of transcriptional co-regulators in DHT-mediated ETS-1 activity was examined. HepG2 cells were co-transfected with SRC-1, AIB-1, NCoR, or SMRT vectors and then treated with 100 nM DHT. As shown in Figure 5D and 5E and Supplementary Figure 7, ETS-1’s androgen-induced activity was enhanced after transfection with SRC-1 or AIB-1 and reduced after transfection with NCoR or SMRT.

### Effect of AR on HepG2 cell proliferation

To decipher whether AR activity modulated the proliferation of HepG2 cells, 3-(4,5-dimethyl-2-thiazolyl)-2,5-diphenyl-2-H-tetrazolium bromide (MTT) and soft agar assays were performed. HepG2 cells were stably transfected with empty vectors, AR vectors, control siRNA, or AR siRNA and cultured in phenol red-free DMEM supplemented with 2% charcoal-stripped FBS added with 100 nM DHT or not. As shown in Supplementary Figure 8, up-regulating AR’s activity increased the HepG2 cells’ proliferative ability. At the same time, overexpression of AR only enhanced the proliferation of HepG2 cells in the presence of DHT; down-regulation of AR expression, in contrast, attenuated the DHT-induced proliferation of HepG2 cells.

**Figure 6:**
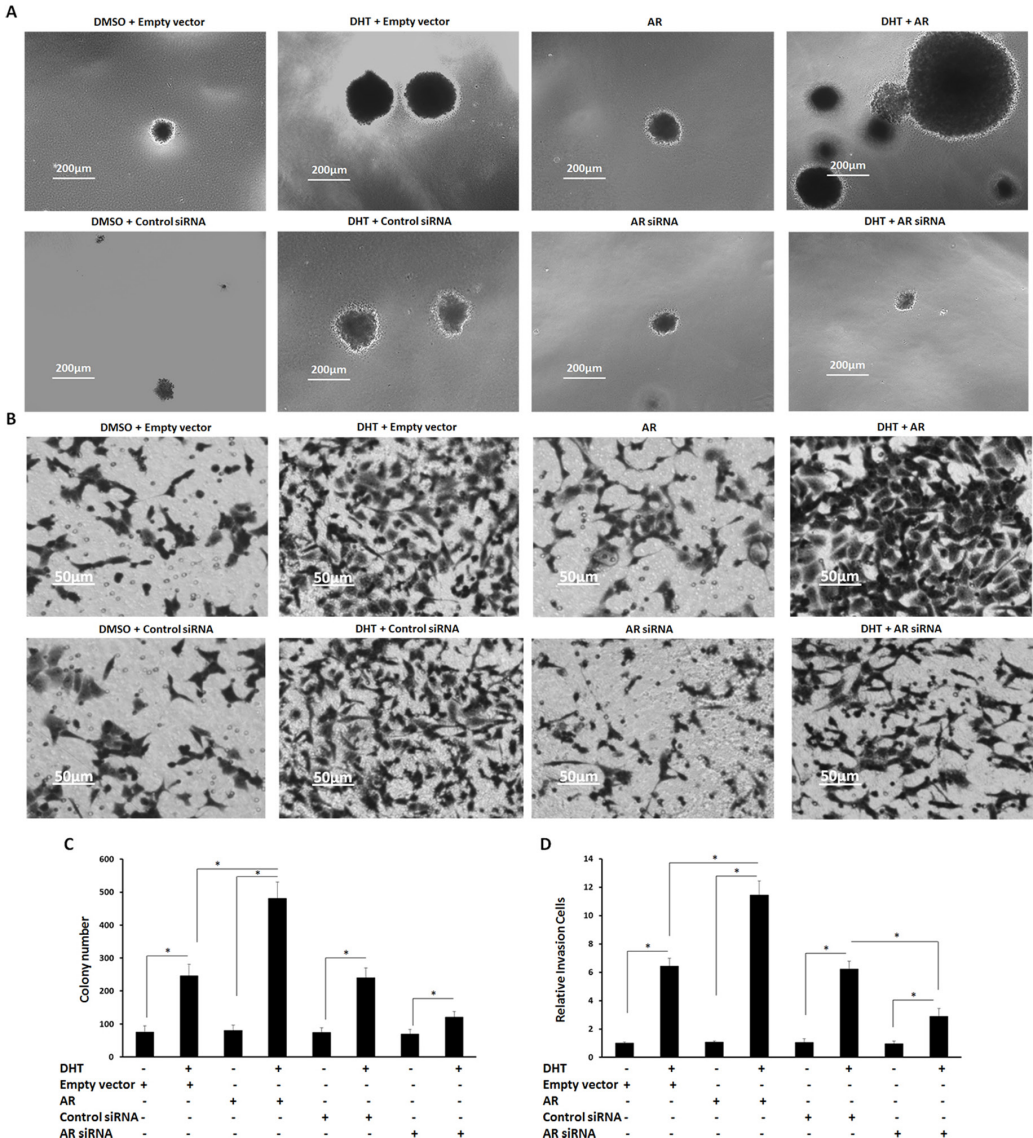
The effect of AR on HepG2 cells’ anchor-independent growth and invasion **(A, B)** HepG2 cells, which were stably transfected with empty vectors, AR vectors, control siRNA, or AR siRNA, were treated with or without 100 nM of DHT. Cells were then measured by soft agar assay (A) or transwell assay (B). Colonies or invasion cells are shown in photographs A and B. **(C, D)** Data are mean ± SD of triplicate independent experiments and were repeated three times with similar numbers. ^*^P < 0.05 versus Solvent control (DMSO) or DHT; ^*^P < 0.05 versus empty vectors or AR vectors.

Next, the effect of AR on HepG2 cell’s anchor-independent growth was examined. Enhancing AR’s activity promoted the anchor-independent growth of HepG2 cells (Figure 6A and 6C) compared with the solvent control (1‰ DMSO). Overexpression of AR only promoted the anchor-independent growth of HepG2 in the presence of DHT. Knockdown of AR’s protein level reduced the DHT-induced anchor-independent proliferation of HepG2 cells (Figure 6A and 6C).

**Figure 7:**
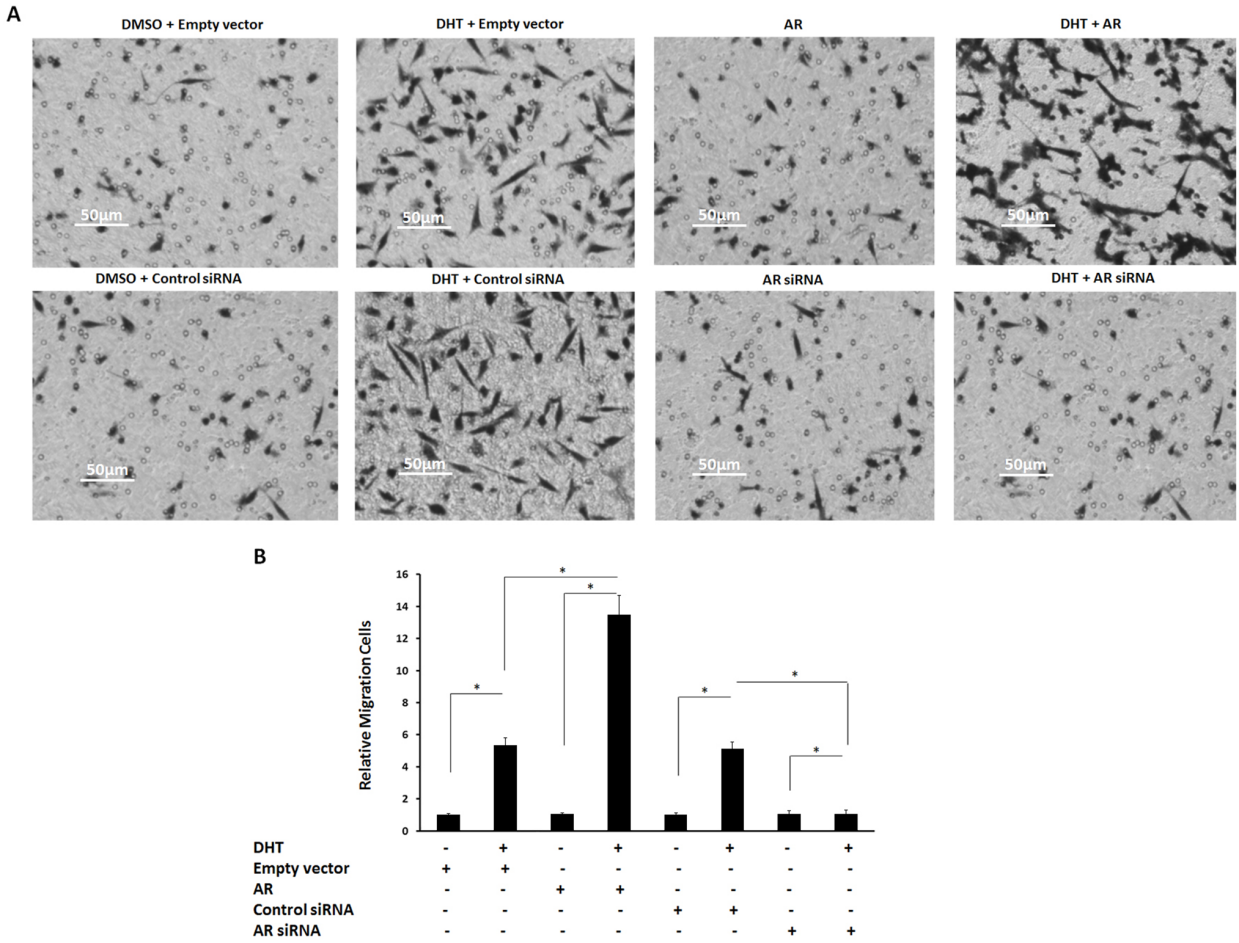
The effect of androgen/AR on HepG2 cells’ migration **(A)** HepG2 cells, which were stably transfected with empty vectors, AR vectors, control siRNA, or AR siRNA, were treated with or without 100 nM of DHT. Then, the cells were measured by transwell assays (A). The migration cells are shown in the photograph (A). **(B)** Mean ± SD of triplicate independent experiments and have been repeated three times with similar numbers. ^*^P < 0.05.

Then, the effect of AR on HepG2 cell’s invasion and migration was also examined. DHT treatment enhanced HepG2 cells’ invasion and migration (Figures 6B and 6D and Figures 7A and 7B), and overexpression of AR only promoted HepG2 cells growth in the presence of DHT. Attenuation of AR’s protein level reduced the effect of DHT on HepG2 cells’ invasion and migration (Figures 6B and 6D and Figures 7A and 7B). These data demonstrate that AR strongly promotes both HepG2 cells’ proliferation and *in vitro* invasion or migration.

**Figure 8:**
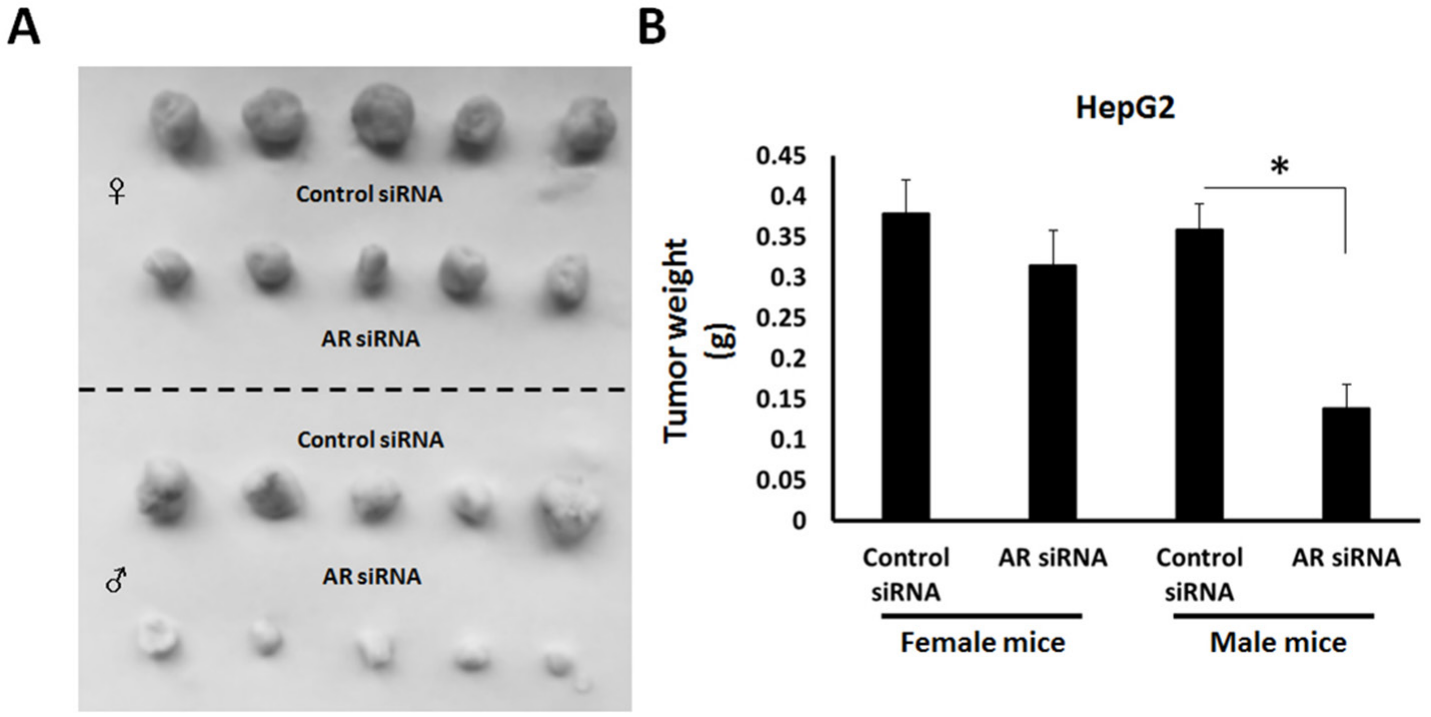
The effect of AR deletion on HepG2 cells’ *in vivo* growth **(A)** HepG2 cells, which were stably transfected with control siRNA or AR siRNA, were seeded in female or male nude mice. Results are shown as photographs (A) or mean ± SD of tumor weight **(B)**. ^*^P < 0.05.

Additionally, the effect of AR deletion on HepG2 cells was tested in nude mice. As shown in Figure 8, transfection with AR siRNA attenuates HepG2 cells’ *in vivo* growth in male mice but not female mice. These results indicate that AR may function via a ligand-dependent manner in HCC.

### Effect of AR on other HCC cells

**Figure 9:**
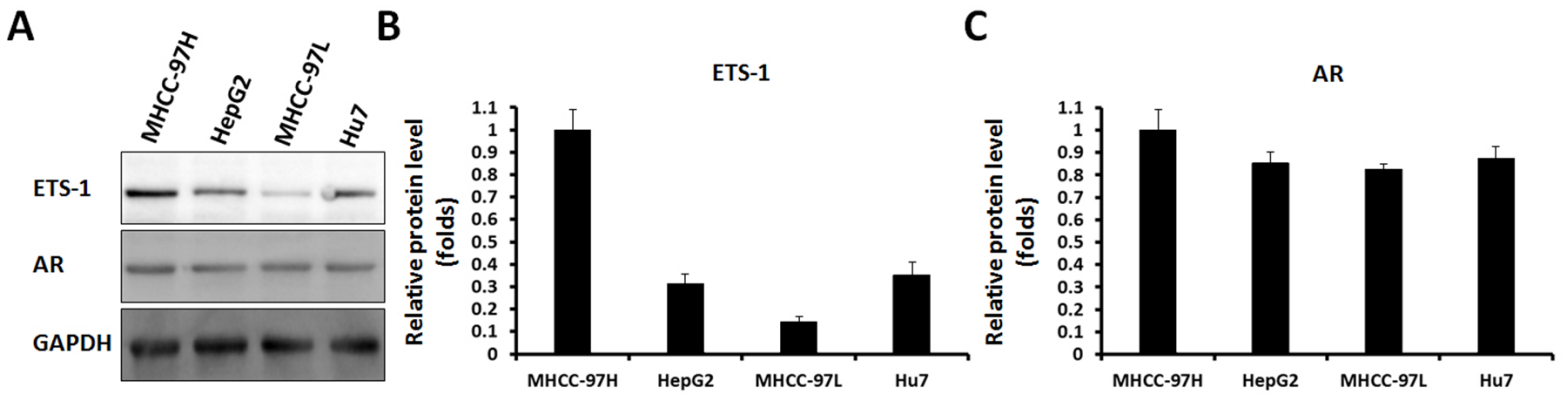
AR and ETS-1 are expressed in HCC cells HCC cells (HepG2, MHCC-97H, Hu7, or MHCC-97L), were harvested for western blot analysis. Results are shown as photographs **(A)** or densitometric analysis **(B** and **C)**. ^*^P < 0.05.

To further confirm the effect of androgen-AR in HCC cells’ proliferation and invasion or migration, other HCC cells were tested. First, the expression of ETS-1 and AR was detected in HCC cells. As shown in Figure 9, a high level of ETS-1 was detected in MHCC-97H cells, a highly aggressive HCC cell line, and a low level of ETS-1 was detected in NHCC-97L cells. Therefore, MHCC-97H was chosen to knock down ETS-1 protein levels and MHCC-97L to overexpression ETS-1.

**Figure 10:**
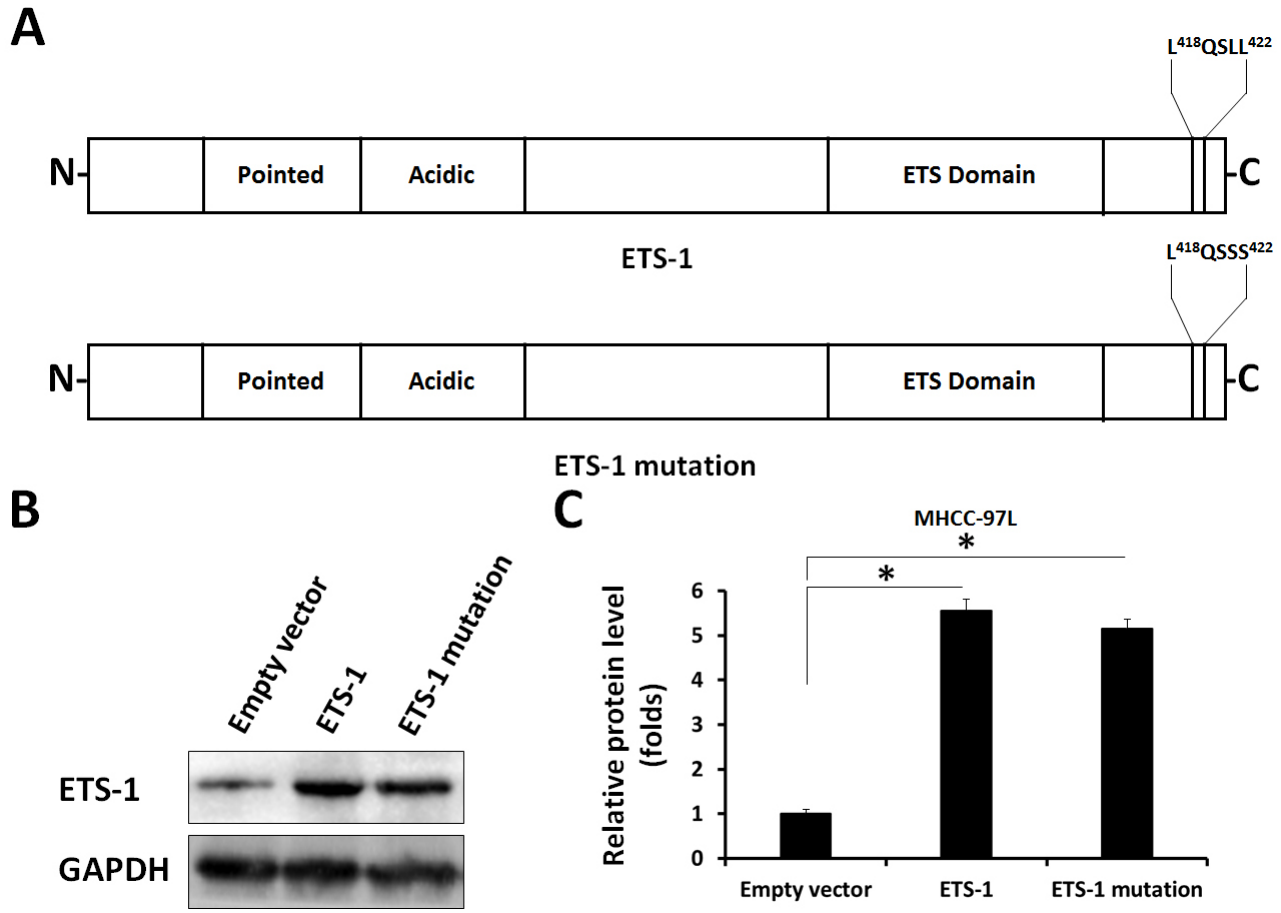
The point mutation of ETS-1 The “LXXLL” motif located in the c-terminal of ETS-1 were mutated. The results are shown as bold, italicized font **(A)**, bands of ETS-1 or GAPDH from western blot **(B)**, or densitometric analysis **(C)**. ^*^P < 0.05.

**Figure 11:**
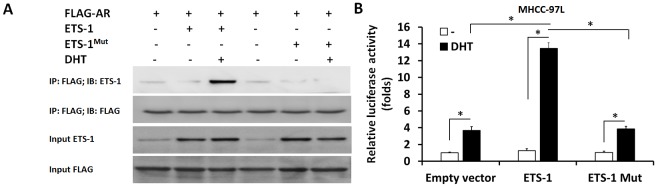
The interaction between AR and ETS-1 or ETS-1 mutation FLAG-tagged AR, wild-type ETS-1, or ETS-1 mutation was transfected into MHCC-97L cells. Cell lysates were immunoprecipitated by an anti-FLAG monoclonal antibody, and the precipitates were then immunoblotted with an anti-ETS-1 or anti-FLAG antibody **(A)**. **(B)** MHCC-97L cells were harvested and analyzed by luciferase. ^*^P < 0.05.

**Figure 12:**
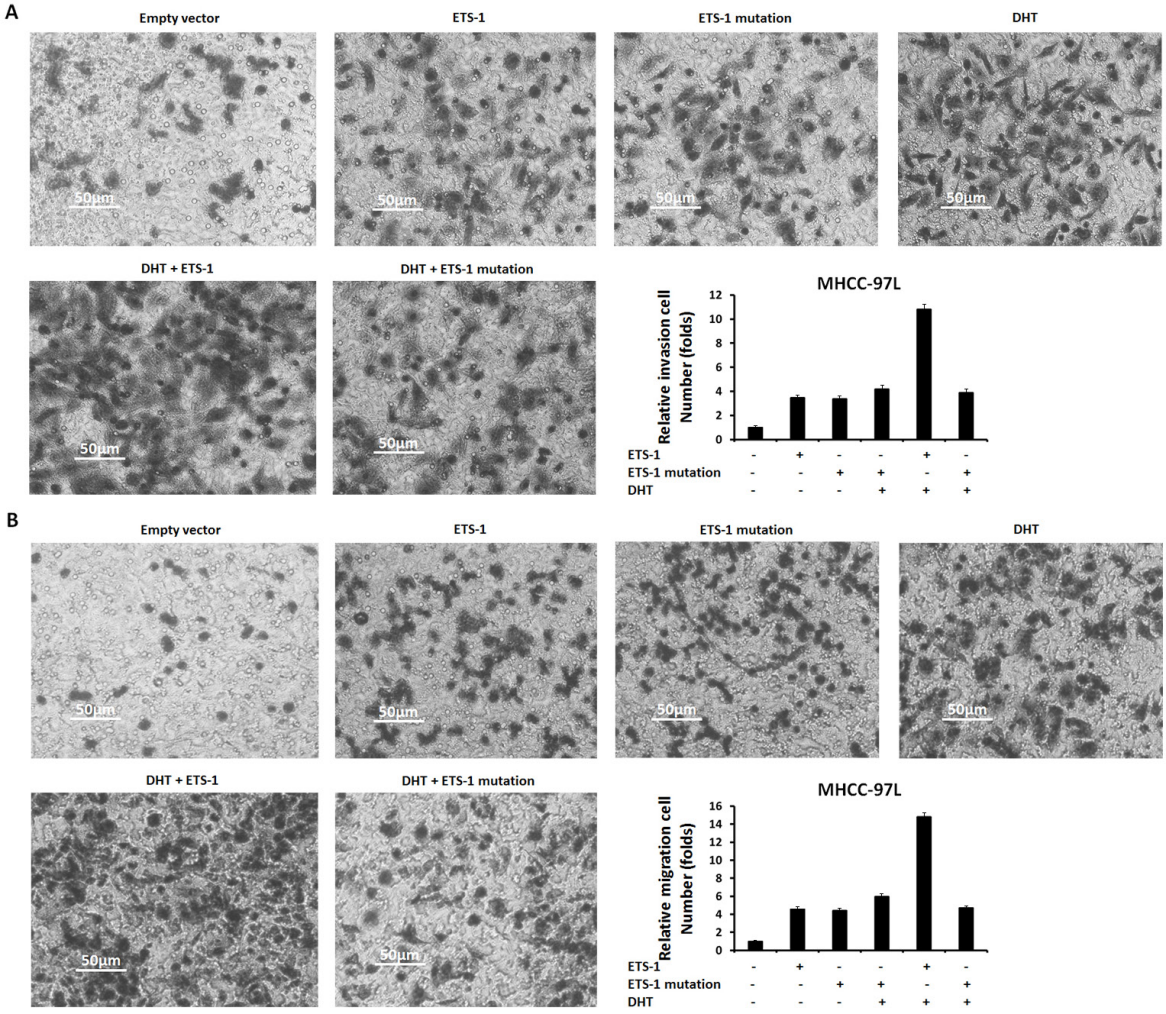
The effect of wild-type ETS-1 or ETS-1 mutation on HepG2 cells’ *in vitro* invasion or migration enhanced by DHT MHCC-97L cells, which were stably transfected with empty vectors, wild-type ETS-1, or ETS-1 mutation vectors, were treated with or without 100 nM of DHT. Then, the cells were measured by transwell assays. The invasion **(A)** or migration **(B)** cells were shown as photographs or mean ± SD of triplicate independent experiments and were repeated three times with similar numbers. ^*^P < 0.05.

**Figure 13:**
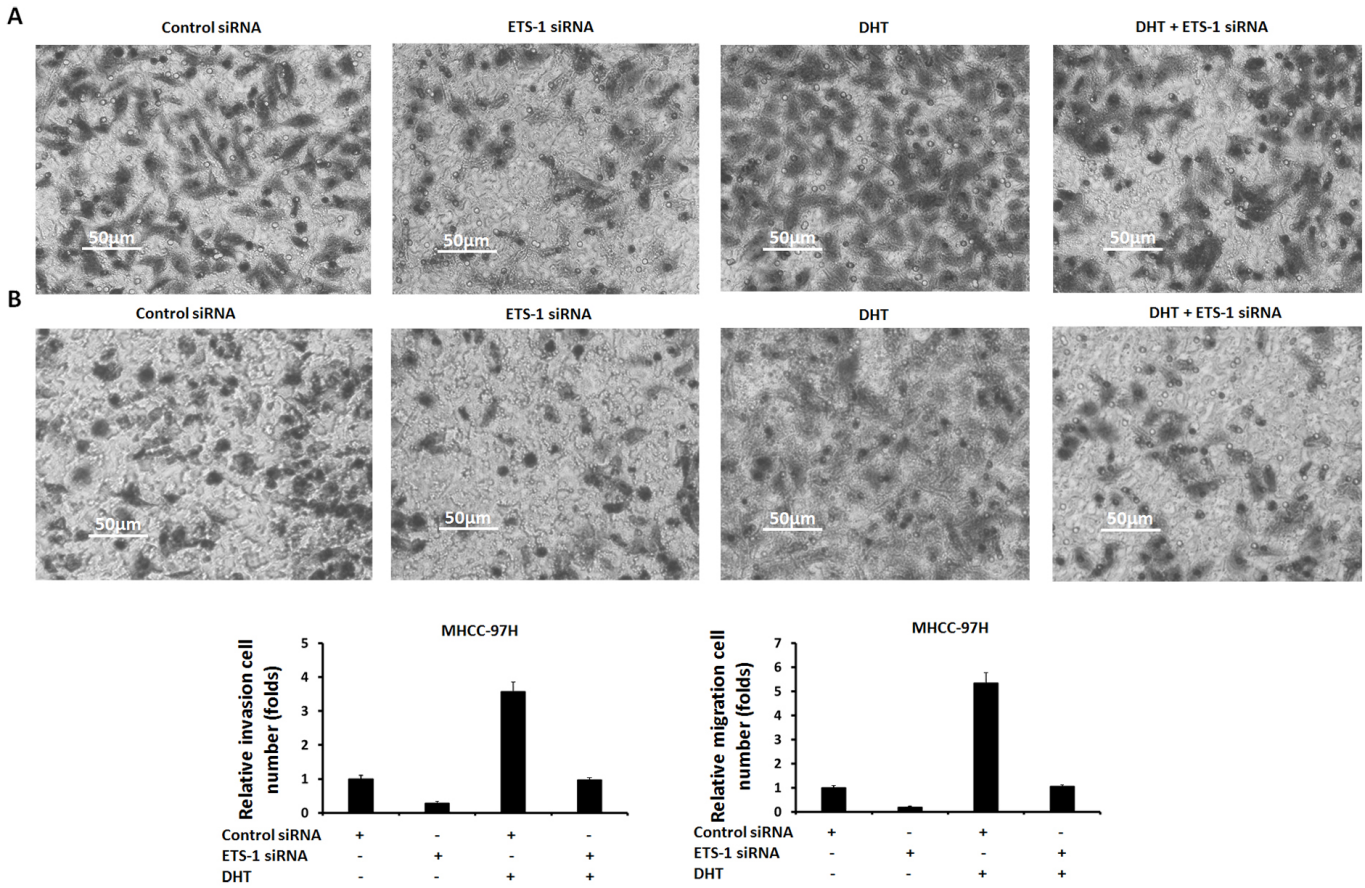
The effect of ETS-1 knockdown on MHCC-97H cells’ *in vitro* invasion or migration enhanced by DHT MHCC-97H cells, which were stably transfected with control siRNA or ETS-1 siRNA, were treated with without 100 nM. Then, the cells were measured by transwell assays. The invasion **(A)** or migration **(B)** cells were shown as photographs or mean ± SD of triplicate independent experiments and were repeated three times with similar numbers. ^*^P < 0.05.

Next, to reveal the specificity of ETS-1 and AR interaction, a point mutation of “LXXLL” motif in ETS-1 C-terminal (Figure 10) was constructed. As shown in Figure 11A and Supplementary Figure 9, only wild-type ETS-1 interacted with AR; ETS-1 mutation did not. Overexpression of wild-type ETS-1 in MHCC-97L cells significantly enhanced the effect of DHT on the EBS-Luc reporter but not on the ETS-1 mutation (Figure 11B). Overexpression of ETS-1 (but not ETS-1 mutation) enhanced the effect of DHT on MHCC-97L cells’ *in vitro* invasion or migration (Figure 12). Moreover, transfection of ETS-1 siRNA in MHCC-97H cells decreased ETS-1 levels and the effect of DHT on EBS-Luc activity or *in vitro* invasion or migration (Figure 13). These results further confirm the interaction between ETS-1 and AR in HCC cells.

## DISCUSSION

This study provides new evidence that AR functions as a novel ETS-1-interacting protein in the presence of androgen. The transcription factor activity of ETS-1 is up-regulated after AR’s activity is enhanced via androgen treatment. Impairment of endogenous AR activity via its antagonist mifepristone reduced ETS-1’s activity induced by AR endogenous agonist DHT. The protein-protein interaction between AR and ETS-1 was validated by GST pull-down or co-IP assay. At the same time, AR promotes HCC cell proliferation, and migration or invasion by interacting with ETS-1 in a ligand-dependent manner, *in vivo* or *in vitro*. This study’s data show that DHT functions by modulating the recruitment of ETS-1 and co-regulators to the EBS sequence of MMP1/9 promoter sequences. The accumulation of ETS-1 in the nucleus would also be increased by DHT treatment. Additionally, the interaction between ETS-1 and AR was also confirmed in HCC cells MHCC-97H and MHCC-97L.

ETS-1, which is a mitogen-activated protein kinase (MAPK)-dependent transcription factor, has been implicated as targeted effector of human epidermal growth factor receptor 2 (HER2) or epidermal growth factor receptor (EGFR) signaling pathway [20]. In the nucleus, ETS-1 mediates the transcription of downstream genes by binding to EBS in response to HGF/c-Met. The HGF/c-Met/ETS-1 pathway mediates the proliferation, development, metastasis, invasion, and angiogenesis of a multitude of human cancer cells [11, 12]. Members of the HGF/c-Met/ETS-1 signaling pathway are expressed in human cancers, and a high level of ETS-1 protein is associated with poor prognosis, disease progression, and metastasis [21]. Previous research identified ETS-1 as a novel therapeutic target of triple negative breast cancer (TNBC), which does not express ER, progesterone receptor (PR), or HER2 and is associated with a significant risk of poor prognosis and metastasis [11, 21]. Earlier research also identified the roles of both AR and ETS-1 in HepG2 proliferation and offered some clinical opportunity for patients suffering from HCC.

At the same time, the transcription factor activity of ETS-1 is regulated by multiple factors. Myers et al. showed that ETS-1 should function by interacting with transcriptional co-regulators such as SRC-1or AIB-1 [9]. Sequence motif LxxLL in Loop 1, located in ETS-1’s ETS domain, has been identified as the recognition site for the SRC/p160 binding region [9–12]. Since AR is a ligand-dependent nuclear receptor/transcriptional factor, AR in the presence of androgen is much more active in stimulating cancerous cells’ proliferation than in the absence of androgen [22, 23]. This study’s results show that AR can efficiently enhance ETS-1’s transcription factor activity in HepG2 cells or AR-negative PC-3 cells stably expressed AR were grown in phenol red-free medium with charcoal dextran-treated FBS only supplemented DHT. Therefore, AR itself is required for ETS-1’s DHT-induced transcription factor activity, and AR may be a novel ETS-1 co-regulator in the presence of androgen. In mammal cells, androgen response to AR, which is arrested in cytosol in the absence of ligand. AR is translocated into the nucleus in the presence of androgen and binds to the genome DNA of the androgen responsive element (ARE) sequences to regulate the expression of targeted genes [2, 3]. This study’s observation that androgen induces the accumulation of ETS-1 in the nucleus and the recruitment of ETS-1 to its targeted genes’ promoter increases the likelihood that AR may interact with ETS-1 to induce its translocation into the nucleus or recruit genes to its DNA-binding sites in the presence of DHT. Further time-effect or dose-effect experiments should be performed to further deduce the mechanisms of androgen/AR on ETS-1’s cytoplasmic/nuclear translocation.

The ETS family also includes many transcription factors or regulatory proteins. All-ETS family members share DNA-binding domains named as ETS domains in the C-terminal part of the protein and similar DNA-binding sites [24] and play compensatory or similar roles in physiological, pharmaceutical, or pathological processes [24]. Thus, the possibility that AR may also interact with other ETS family other members, such as ETS-2, cannot be excluded. Examining the cross-talk of ERα with other members of the ETS family besides ETS-1 is valuable.

Proliferation and invasion or migration are the main features of metastatic malignancies, which explore critical points in cancer progression and are a major cause of mortality. AR contributes to several kinds of human cancer, including prostate cancer, urothelial carcinoma, and especially HCC [15]. AR participates in the regulation of HCC proliferation via several mechanisms. Teng et al. reported that AR enhanced the expression of oncomiR-21 in HCC in the presence of ligand DHE (Dehydroepiandrosterone) [25]. Chen et al. also showed that AR stimulated microRNA-216a expression to suppress tumor suppressors during hepatocarcinogenesis [5]. Results from Jiang et al. indicated that androgen/AR pathway maintains and promotes HCC cells’ stemness [26]. The present study’s data reflect the interaction of AR and ETS-1 and the role of AR in HCC; AR activity was independent of HGF/c-Met signaling. These results are consistent with results from Nie et al. and Zhang et al. [17, 27]. The findings provided in this work reveal AR’s roles in HCC cell proliferation. Some remaining data, however, reveal AR’s tumor-suppressing roles in HCC [28, 29]. Thus, future work should continue to explore AR’s roles in HCC.

Recently, nuclear receptors have been suggested as targets for anti-tumor therapy [30, 31]. Wang et al. and Zhao et al. indicated that AR would be a useful target to enhance the efficacy of sorafenib in HCC treatment [32, 33]. Thus, this study also explored whether the antagonist of AR could be a novel therapeutic strategy for HCC patients.

This study’s results provide important details about AR’s function and mechanism in HCC’s cell proliferation ability. By establishing AR’s roles and mechanisms, this study identified AR as a useful molecular target for HCC therapy.

## MATERIALS AND METHODS

### Plasmids

The expression vectors of ETS-1, FLAG-ETS1, AR, FLAG-AR, AR siRNA, ARE-Luc, MMP1-Luc, and MMP9-Luc were gifts from Dr. Jiajun Cui and Dr. Fan Feng as described in references [2] and [3]. The ETS-1 point mutation sequence was obtained by using chemical synthesis methods and then cloned into a pcDNA3.1 plasmid. The expression vectors of SRC-1 and AIB-1 were purchased from Origene Company, USA. The EBS (GGAA)_8_ sequences were synthesized by using chemical synthesis methods (Gene Ray Company, China) and cloned into a pGL4.26 plasmid. All vectors were confirmed by DNA sequencing. The siRNA of ETS-1 was synthesized by Shanghai GenePharm Company, China. All siRNA were transfected into the cells according to the manufacturer’s protocol.

### Cell cultures and reagents

The ARQ-197 was obtained from Active Biochemicals, Selleck Company, USA. The dihydrotestosterone (DHT, endogenous androgen) and mifepristone (AR’s antagonist) were obtained from Sigma (St. Louis, USA). Compounds were configured to a 10 mM DMSO solution and stored at 4°C. Recombinant human HGF was obtained from Pepro-Tech (Rocky Hill, NJ, USA). The human HCC cell line HepG2, Hu7, MHCC-97H, or MHCC-97L was obtained from the Cell Resources Center of the Chinese Academy of Medical Sciences in China. HepG2 cells were cultured in complete Dulbecco’s modified Eagle’s medium (Invitrogen, Carlsbad, CA) and incubated at 37°C with 5% CO_2_. The human PC-3 prostate cancer cell line was a gift from Dr. Fan Feng and described in reference [2]. PC-3 cells were cultured in an RPMI1640 medium containing 10% FBS (Hyclone, USA).

### Luciferase assay

HepG2 or MHCC-97L cells were seeded in 24-well plates (Corning, NY, USA) containing phenol red-free DMEM supplemented with 0.5% charcoal-stripped FBS (Hyclone, Logan, USA) in the presence or absence of DHT or HGF. PC-3 cells were cultured in an RPMI1640 medium containing 0.5% charcoal-stripped FBS (Hyclone, USA); in the presence or absence of DHT or HGF. Transfection was performed using Lipofectamine 2000 (Invitrogen, Carlsbad, CA). Cells which were co-transfected with luciferase reporters (including EBS-Luc, MMP1/9-Luc, or ARE-Luc, and β-galactosidase loading control) were harvested for luciferase analysis according to the methods described in [34]. The luciferase assays were performed with or without DHT, mifepristone, ARQ-197, or HGF. Similar results were obtained from all three experiments.

### RNA isolation and real-time PCR

The total RNA of the HepG2 cells was extracted using a PARISTM Kit (Applied Biosystems, Foster City, CA) according to the manufacturer’s instructions. A Multiscribe ™ Reverse Transcriptase (Applied Biosystems, Foster City, CA) was used to synthesize the complementary DNA templates. Real-time reverse transcription-polymerase chain reactions were performed by an Applied Biosystems 7500 detection system using Maxima SYBR Green/ROX qPCR Master Mix Assays (Fermentas, Lithuania) and following the protocols provided in reference [3]. The expression of the targeted genes’ mRNA was determined from the threshold cycle (Ct), and the relative expression levels were normalized to the expression of human β-actin mRNA. The primers used in the real-time RT-PCR are listed in Supplementary Table 1.

### Antibodies and western blotting

Total protein samples were performed by SDS-PAGE and incubated with antibodies following the methods provided in reference [35]. Antibodies against AR, ETS-1, MMP1, MMP9, SRC-1, AIB-1, NCoR, SMRT, Lamin A/C, β-actin, and GAPDH were from Santa Cruz Biotechnology, USA. The polyclonal anti-rabbit IgG antibody and anti-Flag monoclonal antibody conjugated with horseradish peroxidase (HRP) were from Sigma, USA. The HepG2 and PC-3 cells, which were stably transfected with plasmids, were seeded and cultured in six-well plates (Corning, NY, USA). The cells, which were treated with the indicated concentration of agents, were harvested by RIPA buffer supplemented with protease inhibitors (Sigma, St. Louis, MO, USA). The total protein samples were performed by SDS-PAGE and transprinted to polyvinylidene fluoride (PVDF) membranes (Millipore, Billerica, MA). Then the membranes were blocked with 10% BSA in TBST buffer and incubated for 2 h at 37°C with a mouse primary antibody targeted to human AR (1:1000), Lamin A/C (1:5000), or β-actin (1:5000); a rabbit primary antibody targeted against ETS-1 (1:2000); a mouse primary antibody targeted against human MMP1 (1:500), MMP9 (1:1000), SRC-1 (1:1000), or AIB-1 (1:1000); a rabbit primary antibody targeted against human NCoR (1:500) or SMRT (1:500); and a mouse primary monoclonal antibody targeted against human GAPDH (1:5000) and diluted in TBST containing 5% BSA and subsequently washed three times in TBST for 5 min each. Then, the blots were incubated with HRP-conjugated secondary antibodies (1:5000) after being washed three times in TBST for 5 min each. Finally, the blot was developed with enhanced chemiluminescence reagents (Pierce, USA) by X-ray films. When the blots were incubating the HRP anti-FLAG monoclonal antibody (1:5000), the blots were visualized without incubating a secondary antibody.

### Immunoprecipitation

HepG2 or MHCC-97L cells were transfected with plasmids using Lipofectamine 2000. Then the cells were harvested and lysed in an immunoprecipitation buffer culture for 18–24 h at 4°C. The Co-IP analysis was performed with an anti-FLAG monoclonal antibody (Sigma-Aldrich, USA) and then detected by immunoblotting assays treated without or with 100 nM DHT following the protocol described in reference [3].

### GST-pull down

GST-AR or GST-ETS1 was expressed in E. coli strain DH5α and bound to glutathione-Sepharose beads purified as described by the manufacturer (Amersham Biosciences, Milpitas, USA). FLAG-AR or FLAG-ETS1 was expressed in HEK293T cells and purified by FLAG-beads. FLAG-AR or FLAG-ETS1 was incubated with GST alone, GST-ETS1, or GST-AR bound to glutathione-Sepharose beads from reference [36] at 4°C. The beads were precipitated, washed three times with binding buffer, and subjected to SDS-PAGE and western blotting.

### Subcellular fractionation

The subcellular localization of AR and ETS-1 was determined by subcellular fractionation assays following the protocol described in reference [16]. Cells were homogenized using a Dounce homogenizer, and the homogenate was centrifuged at 366 g for 10 min. The pellet was analyzed as the nuclear fraction. The supernatant was centrifuged again at 13,201 g for 15 min, and the final supernatant was analyzed as the cytoplasmic fraction. The β-actin was the cytoplasm’s indicator, and Lamin A/C was used as the nuclear indicator.

### Chromatin immunoprecipitation

The chromatin immunoprecipitation (ChIP) was performed following the protocol provided by the ChIP kit (Upstate, NY, USA) and as described in reference [16]. HepG2 cells were fixed by adding formaldehyde to the medium to a final concentration of 1%. After cross-linking, glycine was added to a final concentration of 125 mM, and the cells were then harvested with lysis buffer. The nuclear part of the cells was pelleted by centrifugation, resuspended in nuclear lysis buffer, and sonicated to generate DNA fragments. Then the immunoprecipitation assay was performed with antibodies. Real-time PCR was performed with DNA extracted from the immunoprecipitates and primers flanking the EBS in ETS-1 targeted gene’s promoter. The primers are listed in Supplementary Table 2.

### Cell proliferation assay

Cell proliferation was analyzed by MTT-assay as described previously in reference [3]. HepG2 cells were cultured in phenol red-free DMEM supplemented with 2% charcoal-stripped FBS and with or without added DHT. Then, the proliferation of HepG2 cells was determined using a Cell Titer 96^®^ nonradioactive cell proliferation assay kit (Promega, USA), per the manufacturer’s instructions. Cells, which were transfected with plasmids or treated with agents, were seeded onto 96-well plates and incubated at 37°C with 5% CO_2_. After incubating for 24, 48, 72, 96, 120, and 144 h, cells were harvested and analyzed. Finally, growth curves for each cell group were drawn according to the volume of *O.D.*_*490*_ nm values from the 96-well plate reader. The MTT cell growth assays were performed three times independently.

### Anchorage-independent growth

HepG2 cells were treated with compounds. Cells were plated on six-well plates (500 per well), with a bottom layer of 0.7% low-melting-temperature agar in phenol red-free DMEM supplemented with 2% charcoal-stripped FBS and a top layer of 0.25% agar in phenol red-free DMEM supplemented with 2% charcoal-stripped FBS. The colony number was the mean ± SD of triplicate independent experiments scored after 3–4 weeks of growth [37].

### Transwell assay

The invasion and migration assays were performed in 24-well plates using a transwell chamber (Corning, NY, USA) fitted with a polyethylene terephthalate filter membrane with 8-μm pores following the protocol described in [38]. For the invasion assay, the membrane undersurface was coated with 30μl of ECM gel extracted from Engelbreth-Holm-Swarm mouse sarcoma (BD Biosciences, Bedford, MA, USA) mixed with RPMI-1640 serum free medium in 1:5 dilution for 4 h at 37°C. The top chambers of the transwells were filled with 0.2 ml of cells (5 × 10^5^ cells/ml) in serum-free medium, and the bottom chambers were filled with 0.25 ml of an RPMI 1640 medium containing 10% FBS. The cells were incubated in the transwells at 37°C in 5% CO_2_ for 4 h or 24 h. The relative invading/migrating cells were measured following the methods described previously in [38].

### Statistical analysis

The results of the RT-PCR and WB assays were analyzed by ALPHA INNOTECH software. The relative expression level [39] = (indicated group expression level / loading control expression level) / (control group expression level / loading control expression level). All statistical significance analyses were performed by Bonferroni correction with or without two-way ANOVA using SPSS statistical software. *P* < 0.05 was considered statistically significant. The *EC*_*50*_/*IC*_*50*_ values were calculated by Origin 6.0 software.

## SUPPLEMENTARY MATERIALS FIGURES AND TABLES


