# Supplementary material for: Androgen enhances the activity of ETS-1 and promotes the proliferation of HCC cells
Source: Oncotarget. 2017 Nov 25;8(65):109271–88. doi: 10.18632/oncotarget.22669 (PMC5752520; doi:10.18632/oncotarget.22669)
Supplement: Supplementary file 1 [file oncotarget-08-109271-s001.pdf]

Androgen enhances the activity of ETS-1 and promotes the proliferation of HCC cells

SUPPLEMENTARY MATERIALS

Supplementary Table 1: Real-time RT-PCR primers

Target genes	Primers
MMP1	Forward primer: 5’-aagccatcacttaccttgcact-3’
	Reverse primer: 5’-tcagagaccttggtgaatgtca-3’
MMP9	Forward primer: 5’-ctggagacctgagaaccaa-3’
	Reverse primer: 5’-actgctcaaagcctccacaaga-3’
β-Actin	Forward primer: 5’-ctccatcctggcctcgctgt-3’
	Reverse primer: 5’-gctgtcaccttcaccgttcc-3’

Supplementary Table 2: Primers used in ChIP assays

Target gene	Primers
Input	Forward: 5’-AACCTATTAACTCACCCTTGT-3’
	Reverse: 5’-CCTCCATTCAAAAGATCTTATTATTTAGCATCTCCT-3’
MMP1	Forward: 5’- TTCCAGCCTTTTCATCATCC-3’
	Reverse: 5’- CGGCACCTGTA CTGACTGAA -3’
MMP9	Forward: 5’- TACATTGGTACCTCTTGGGTCTTGGCCTTAGT-3’
	Reverse: 5’- TTGATACTCGAGCCAGCACCAGGAGCACC-3’

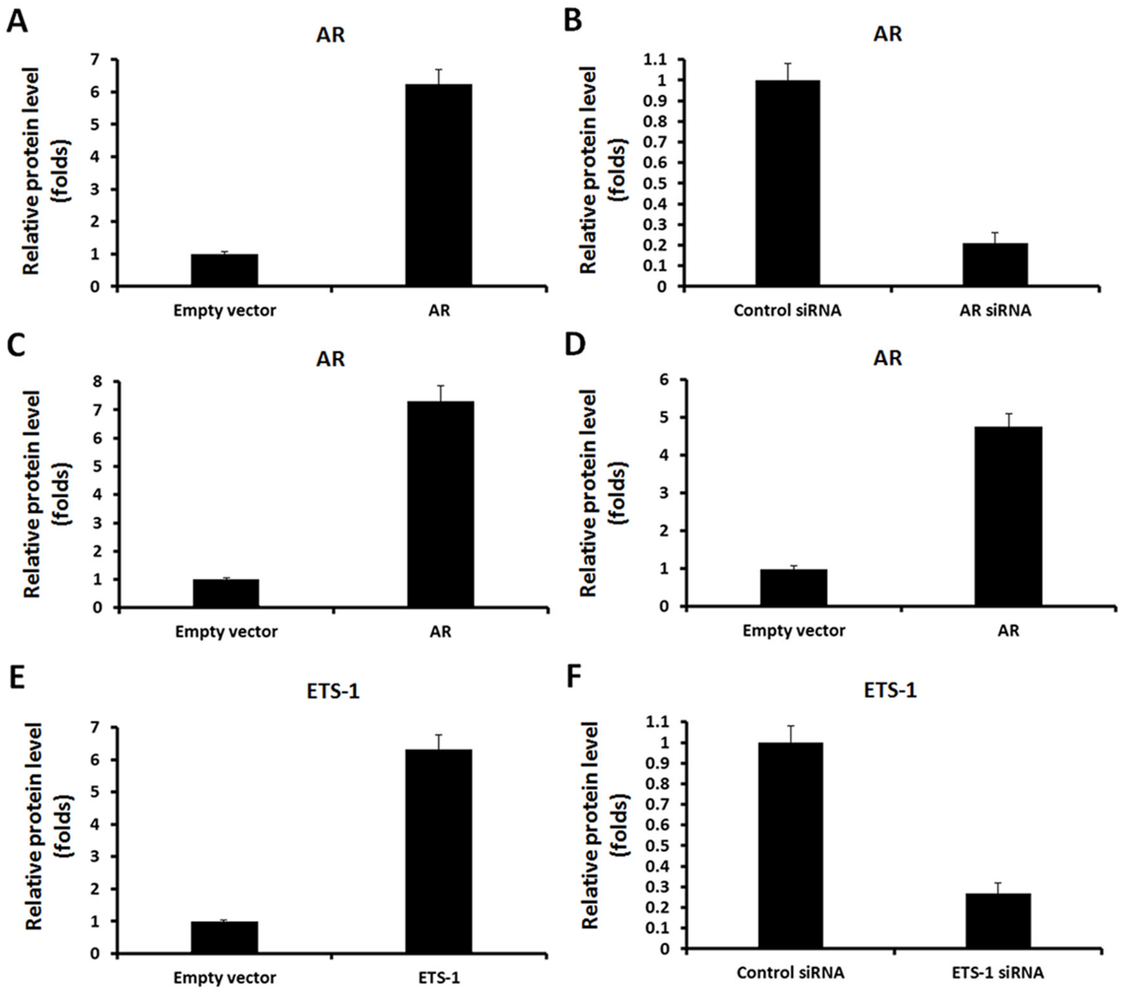

Supplementary Figure 1: The densitometric analysis of the western blot data in Figure 3.

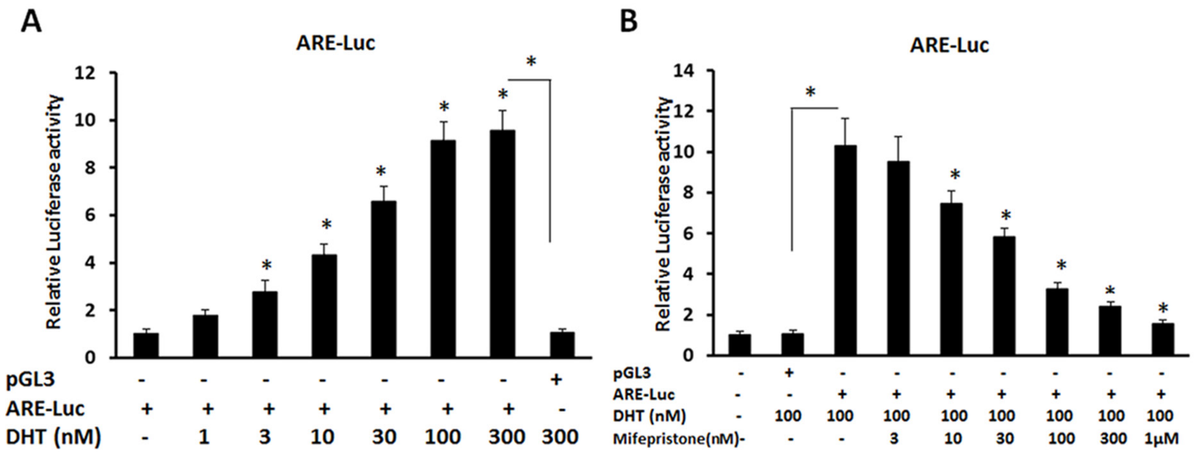

Supplementary Figure 2: The transcription factor activity of AR in PC-3 cells. PC-3 cells, which were co-transfected with ARE-Luc reporters or AR vectors, were treated with indicated concentration **(A** and **B)** of DHT or (B) Mifepristone. Then, cells were harvested and determined by the Luciferase assays. The values are the mean±SD from three independent experiments. * P<0.05.

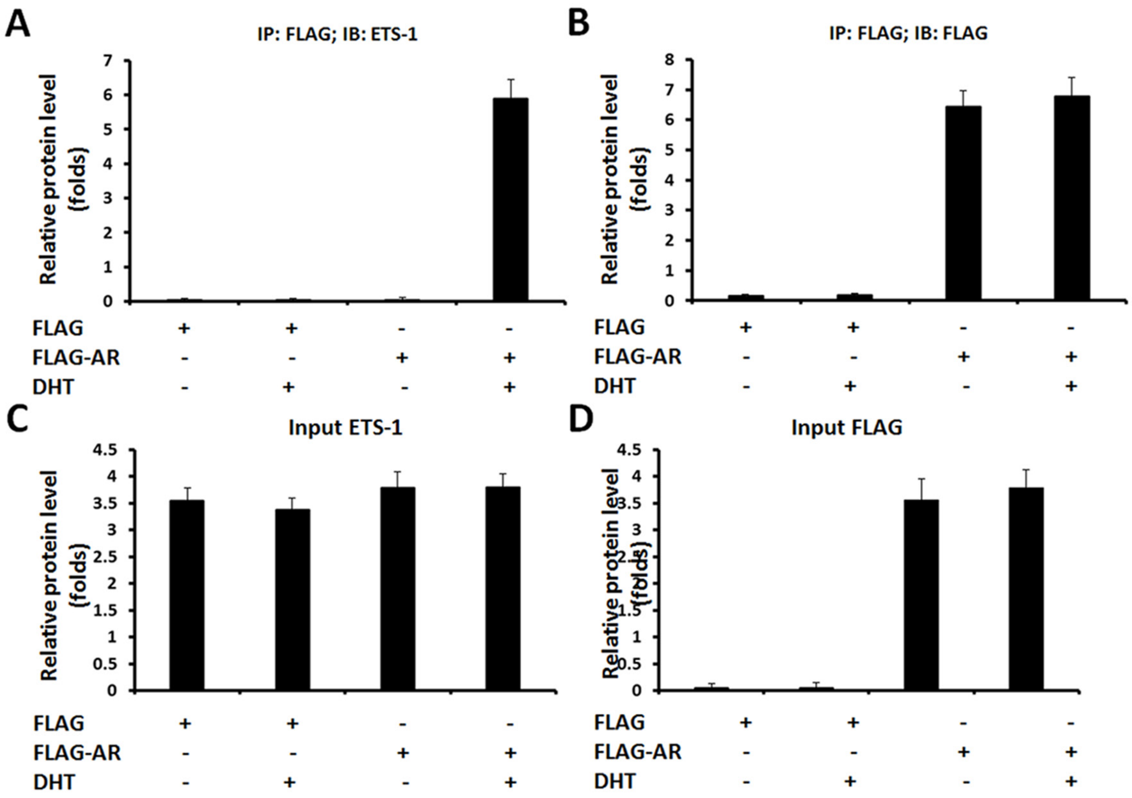

Supplementary Figure 3: The densitometric analysis of the western blot data in Figure 4A.

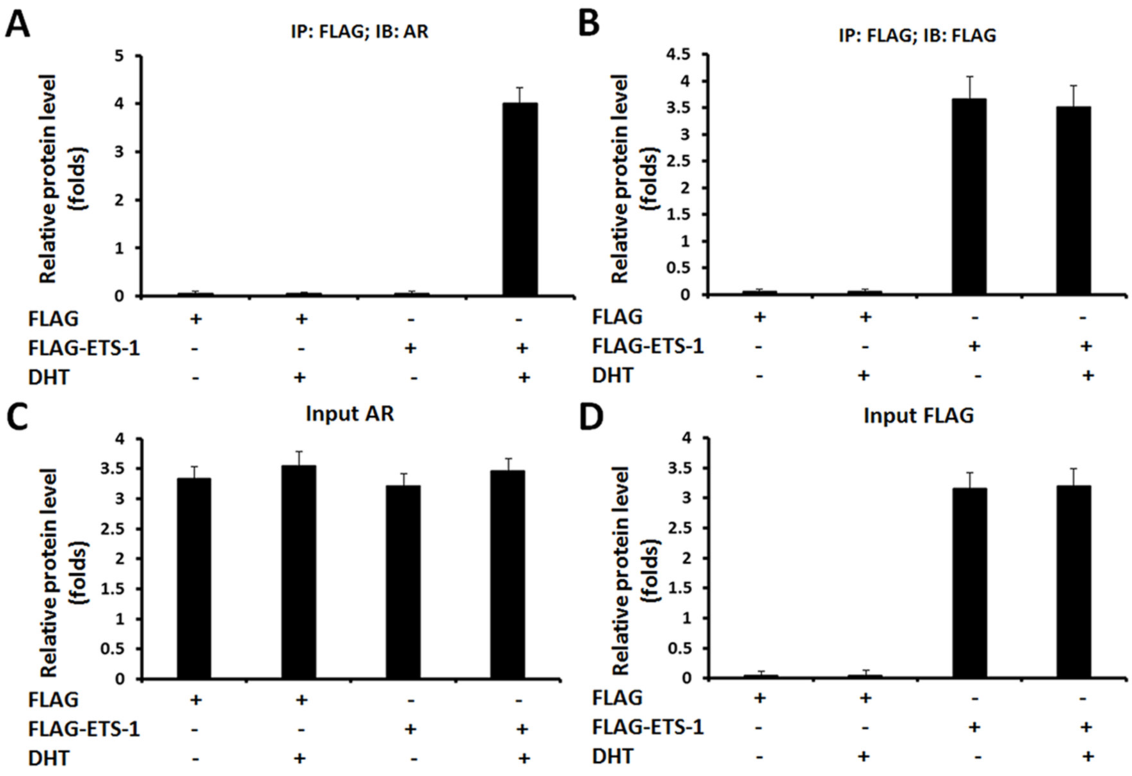

Supplementary Figure 4: The densitometric analysis of the western blot data in Figure 4B.

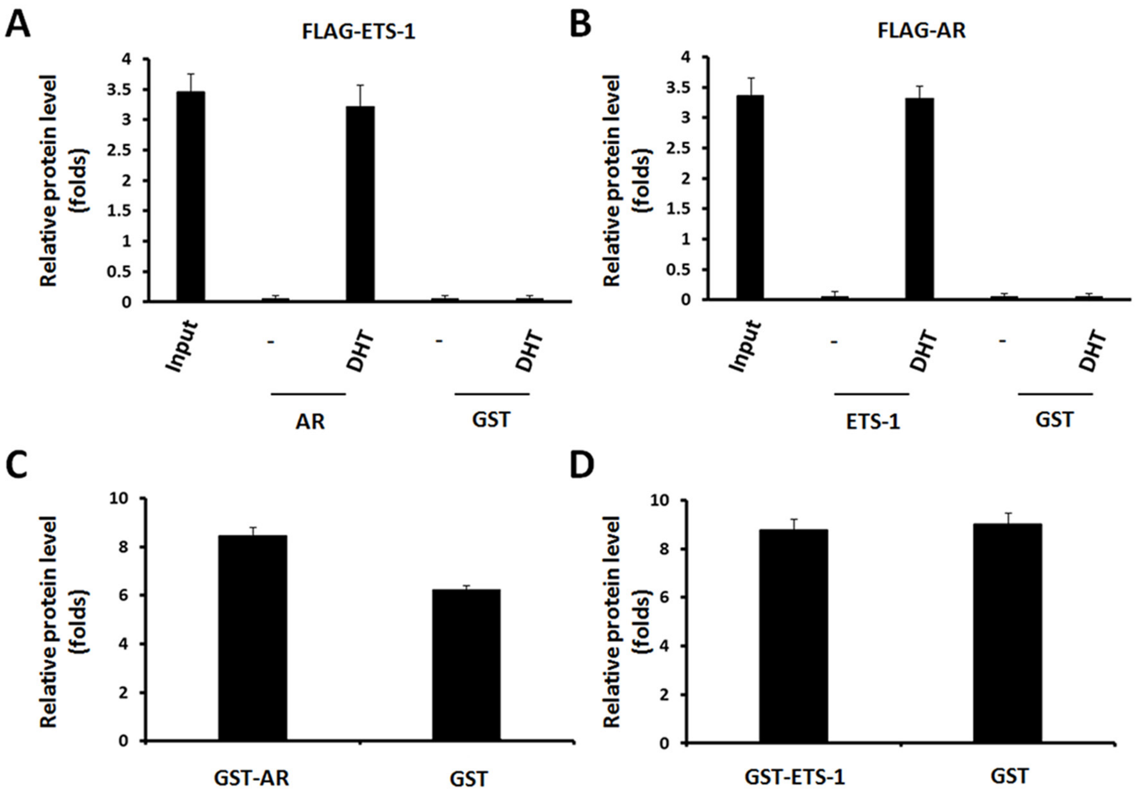

Supplementary Figure 5: The densitometric analysis of the western blot data in Figure 4C and 4D.

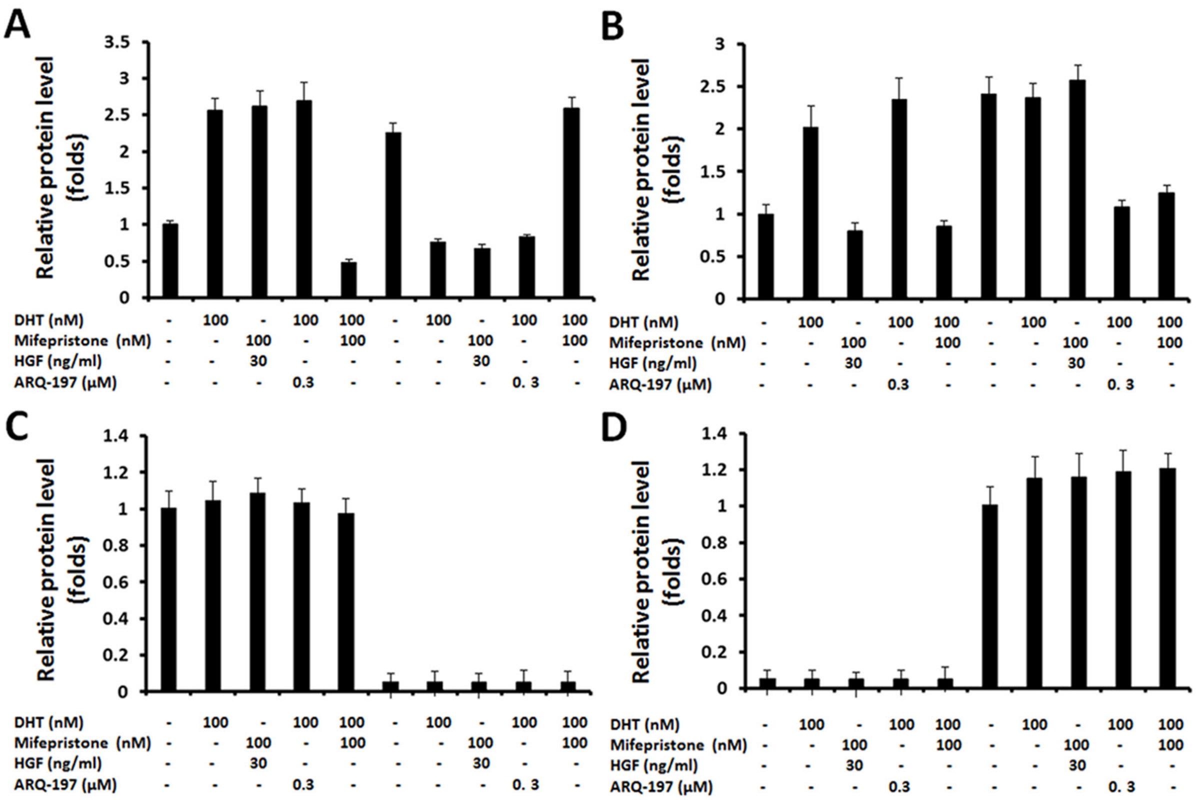

Supplementary Figure 6: The densitometric analysis of the western blot data in Figure 5A.

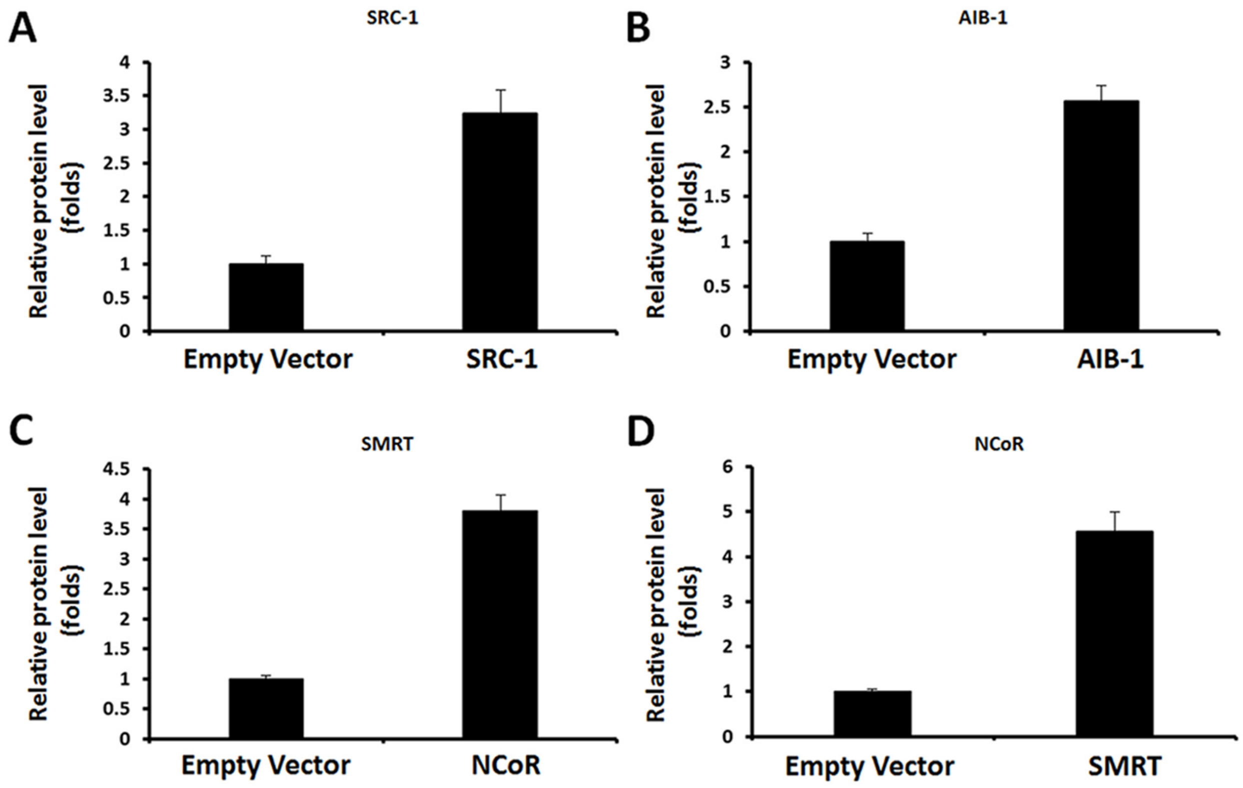

Supplementary Figure 7: The densitometric analysis of the western blot data in Figure 5D-5E.

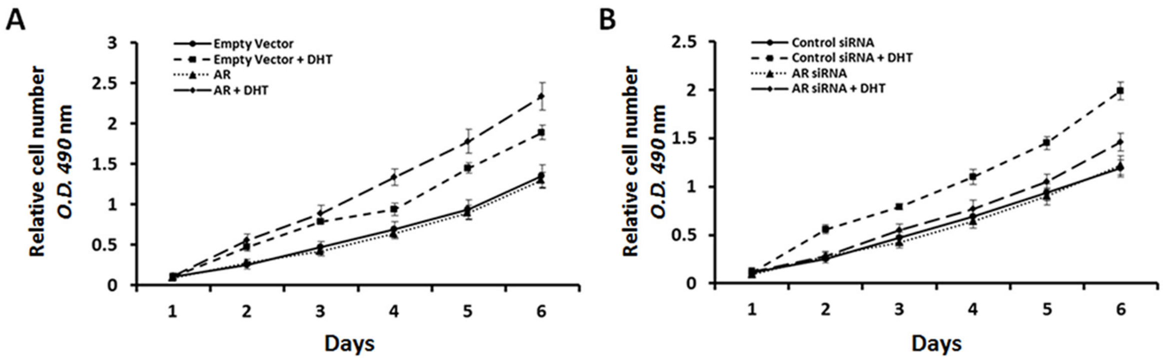

Supplementary Figure 8: Effect of AR on HepG2 cells' proliferation. **(A, B)** HepG2 cells, which were stably transfected with empty vectors (A), AR vectors (A), control siRNA (B) or AR siRNA (B), were treated with DHT (100nM) or not. Cells were then measured by MTT assays. Data are mean ± SD of triplicate independent experiments and have been repeated 3 times with similar numbers.

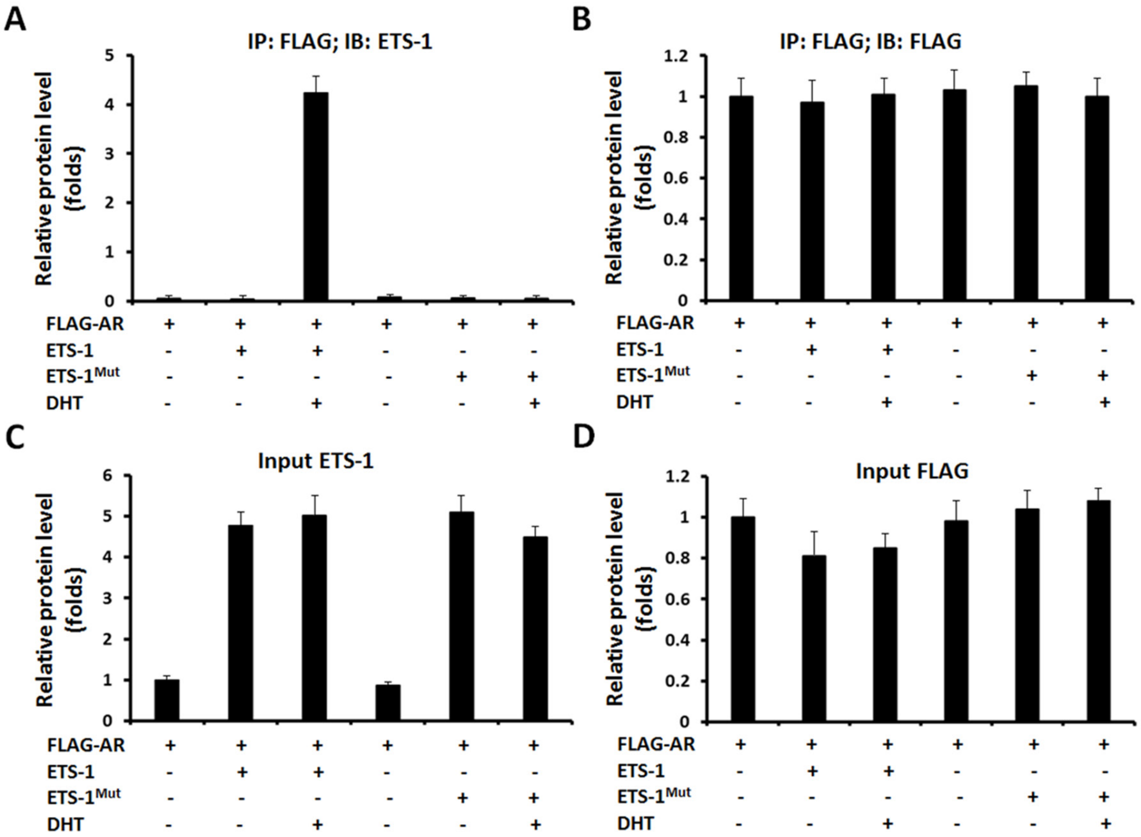

Supplementary Figure 9: The densitometric analysis of the western blot data in Figure 11A.
